# Salt-inducible kinase 2 regulates fibrosis during bleomycin-induced lung injury

**DOI:** 10.1016/j.jbc.2022.102644

**Published:** 2022-10-26

**Authors:** Manuel van Gijsel-Bonnello, Nicola J. Darling, Takashi Tanaka, Samuele Di Carmine, Francesco Marchesi, Sarah Thomson, Kristopher Clark, Mariola Kurowska-Stolarska, Henry J. McSorley, Philip Cohen, J. Simon C. Arthur

**Affiliations:** 1 Division of Cell Signalling and Immunology, School of Life Sciences, University of Dundee, Dundee, United Kingdom; 2 MRC Protein Phosphorylation and Ubiquitylation Unit, School of Life Sciences, University of Dundee, Dundee, United Kingdom; 3 Research Centre of Specialty, Ono Pharmaceutical Co Ltd, Osaka, Japan; 4 School of Veterinary Medicine, College of Medical Veterinary and Life Sciences, University of Glasgow, Glasgow, United Kingdom; 5 Biological Services, University of Dundee, Dundee, United Kingdom; 6 Institute of Infection, Immunity and Inflammation, College of Medical Veterinary and Life Sciences, University of Glasgow, Glasgow, United Kingdom

**Keywords:** SIK2, SIK1, SIK3, pulmonary fibrosis, nintedanib, kinase inhibitor, BALF, bronchoalveolar lavage fluid, BMDM, bone marrow–derived macrophage, CREB, cAMP response element–binding protein, CRTC, CREB-regulated transcriptional coactivator, CI, confidence interval, FBS, fetal bovine serum, FGFR, fibroblast growth factor receptor, IL, interleukin, LPS, lipopolysaccharide, M-CSF, macrophage colony-stimulating factor, PDGFR, platelet-derived growth factor receptor, SIK, salt-inducible kinase, TNF, tumor necrosis factor, VEGFR, vascular endothelial growth factor receptor

## Abstract

Idiopathic pulmonary fibrosis is a progressive and normally fatal disease with limited treatment options. The tyrosine kinase inhibitor nintedanib has recently been approved for the treatment of idiopathic pulmonary fibrosis, and its effectiveness has been linked to its ability to inhibit a number of receptor tyrosine kinases including the platelet-derived growth factor, vascular endothelial growth factor, and fibroblast growth factor receptors. We show here that nintedanib also inhibits salt-inducible kinase 2 (SIK2), with a similar IC_50_ to its reported tyrosine kinase targets. Nintedanib also inhibited the related kinases SIK1 and SIK3, although with 12-fold and 72-fold higher IC_50_s, respectively. To investigate if the inhibition of SIK2 may contribute to the effectiveness of nintedanib in treating lung fibrosis, mice with kinase-inactive knockin mutations were tested using a model of bleomycin-induced lung fibrosis. We found that loss of SIK2 activity protects against bleomycin-induced fibrosis, as judged by collagen deposition and histological scoring. Loss of both SIK1 and SIK2 activity had a similar effect to loss of SIK2 activity. Total SIK3 knockout mice have a developmental phenotype making them unsuitable for analysis in this model; however, we determined that conditional knockout of SIK3 in the immune system did not affect bleomycin-induced lung fibrosis. Together, these results suggest that SIK2 is a potential drug target for the treatment of lung fibrosis.

Pulmonary fibrosis is a serious condition characterized by scarring of the lungs because of excessive extracellular matrix deposition that occurs over time, leading to an irreversible reduction in lung function. It has been associated with several risk factors including exposure to silica or asbestos dust, infection, autoimmune disease, ionizing radiation, and some chemotherapy drugs. Idiopathic pulmonary fibrosis (IPF) is a form of pulmonary fibrosis for which there is no clear cause and is estimated to account for approximately 20% of interstitial lung disease (reviewed in Refs. (1, 2, 3, 4)). The global prevalence of IPF has recently been reported to be between 0.33 and 4.51 cases per 10,000 (1), and the median age at diagnosis is around 65 years old (2, 3, 5). Disease progression is variable, and the reported median survival times from diagnosis range from 2 to 4 years. Treatments for IPF are limited but include pulmonary rehabilitation, supplemental oxygen, and two disease-modifying drugs, nintedanib and pirfenidone. These may slow disease progression but are not curative, and so improved therapies are needed (2, 3, 5).

The molecular mechanisms underlying the development of IPF are complex and not fully understood. It is proposed that repeated microinjuries to the epithelia results in an excessive tissue repair response that leads to the formation of fibroblastic loci and the differentiation of myofibroblasts that result in excess deposition of extracellular matrix (2, 3, 5). This process is accompanied by inflammation; however, it has been debated if inflammation is critically involved in driving disease pathology or if it is a secondary phenomenon. Classical immunosuppressive therapies have proven to be ineffective in IPF; however, this does not exclude a more nuanced role for distinct subsets of immune cells (4). One immune cell that has been proposed to play a role is the macrophage. Macrophages are plastic cells, and their roles vary depending on context; early in inflammation, macrophages adopt a proinflammatory phenotype, sometimes termed “M1” or classically activated macrophages. In contrast, during resolution or tissue repair, macrophages can adopt an anti-inflammatory and potentially profibrotic phenotype. This was originally described in response to activation by the Th2 cytokine interleukin-4 (IL-4) and termed “M2a” or alternatively activated macrophages. This “M2a”-like activation has been linked to fibrosis in different pathologies (6, 7), including IPF (8, 9).

At present, there is no single animal model that fully recapitulates all the features of IPF. However, several models, including bleomycin-induced fibrosis, have been used to evaluate potential targets and therapies in a preclinical setting (10). Bleomycin, an antibiotic made by *Streptococcus verticillus*, has been used as a chemotherapeutic agent in several conditions, including head and neck squamous cell carcinomas, testicular carcinomas, ovarian cancer, and lymphomas (11). Its clinical use is however limited by lung toxicity, and it can cause fibrosis reminiscent of that found in interstitial lung diseases including IPF; this observation led to its use in rodents as a lung fibrosis model (12). Multiple studies have implicated a role for macrophages in bleomycin-induced lung injury. For example, depletion of macrophages with clodronate reduces bleomycin-induced lung fibrosis (8, 13), whereas macrophage colony-stimulating factor 1 (M-CSF1) knockout mice (which have greatly reduced macrophage numbers) show a similar protection (14). More recently, it has been suggested that the differentiation of monocyte-derived alveolar macrophages, rather than pre-existing tissue resident alveolar macrophages, is required for bleomycin-induced fibrosis (15, 16). Knockout of proteins that promote an M2/IL-4-like activation phenotype has been reported to protect against bleomycin-induced fibrosis and reduce expression of markers associated with IL-4-induced activation of macrophages in the lung (17, 18, 19, 20, 21). Furthermore, myeloid-specific knockout of the phosphatases PTEN and Shp2 resulted in increased fibrosis and promoted an M2/IL-4-like activation state in macrophages (22, 23).

Salt-inducible kinases (SIKs) form a subgroup of the calmodulin-dependent kinase subfamily of protein kinases, and three isoforms, SIK1, SIK2, and SIK3, exist in mammalian cells (24, 25, 26). One of the main physiological functions ascribed to SIKs so far is the regulation of cAMP response element–binding protein (CREB)–dependent gene transcription. SIKs phosphorylate the CREB-regulated transcriptional coactivators (CRTCs), resulting in their binding to 14-3-3 proteins and retention in the cytoplasm (24, 25, 27, 28). Inhibition of SIKs results in the dephosphorylation of CRTCs, allowing their translocation to the nucleus, where they interact with CREB to promote the transcription of CREB-dependent immediate early genes. In innate immune cells, such as macrophages, CREB is required for the efficient transcription of the anti-inflammatory cytokine IL-10 (29, 30). In line with this, inhibition of SIKs promotes IL-10 production in macrophages. In addition, SIKs repress the production of proinflammatory cytokines, such as tumor necrosis factor (TNF) (31, 32). SIK2 and SIK3 are the major SIK isoforms expressed in macrophages (33). Macrophages with a kinase-inactive knockin mutation in SIK2 or SIK3 produced elevated levels of IL-10 and lower levels of proinflammatory cytokines in response to Toll-like receptor agonists compared with wildtype macrophages. SIK-inactive knockin macrophages also showed elevated levels of mRNA encoding Sphk1, Arg1, and Light, genes associated with an “M2b-like” proresolution phenotype in macrophages (33).

The shift in macrophages to a proresolution phenotype in SIK knockin mice suggests that SIK inhibitors may act as immunomodulatory drugs. Interestingly, several clinically approved tyrosine kinase inhibitors including dasatinib and bosutinib, have been reported to also inhibit SIKs (34). Tyrosine kinase inhibitors have attracted attention as antifibrotic treatments with several showing protective effects in animal models of lung fibrosis (reviewed in Refs. (35, 36)). One of these, nintedanib, was successful in phase 3 trials for IPF (37) and gained Food and Drug Administration approval for this disease in 2014 (38, 39). The finding that dasatinib protects against bleomycin-induced lung fibrosis in mice (40) together with its ability to inhibit SIKs and the immune-modulatory functions of SIKs led us to investigate the role of SIKs in lung fibrosis. We show here that loss of SIK2 activity protects against bleomycin-induced lung fibrosis in mice, and that the clinically approved IPF drug nintedanib is a potent inhibitor of SIK2.

## Results

### Nintedanib inhibits SIK2 *in vitro* and in cells

**Figure 1:**
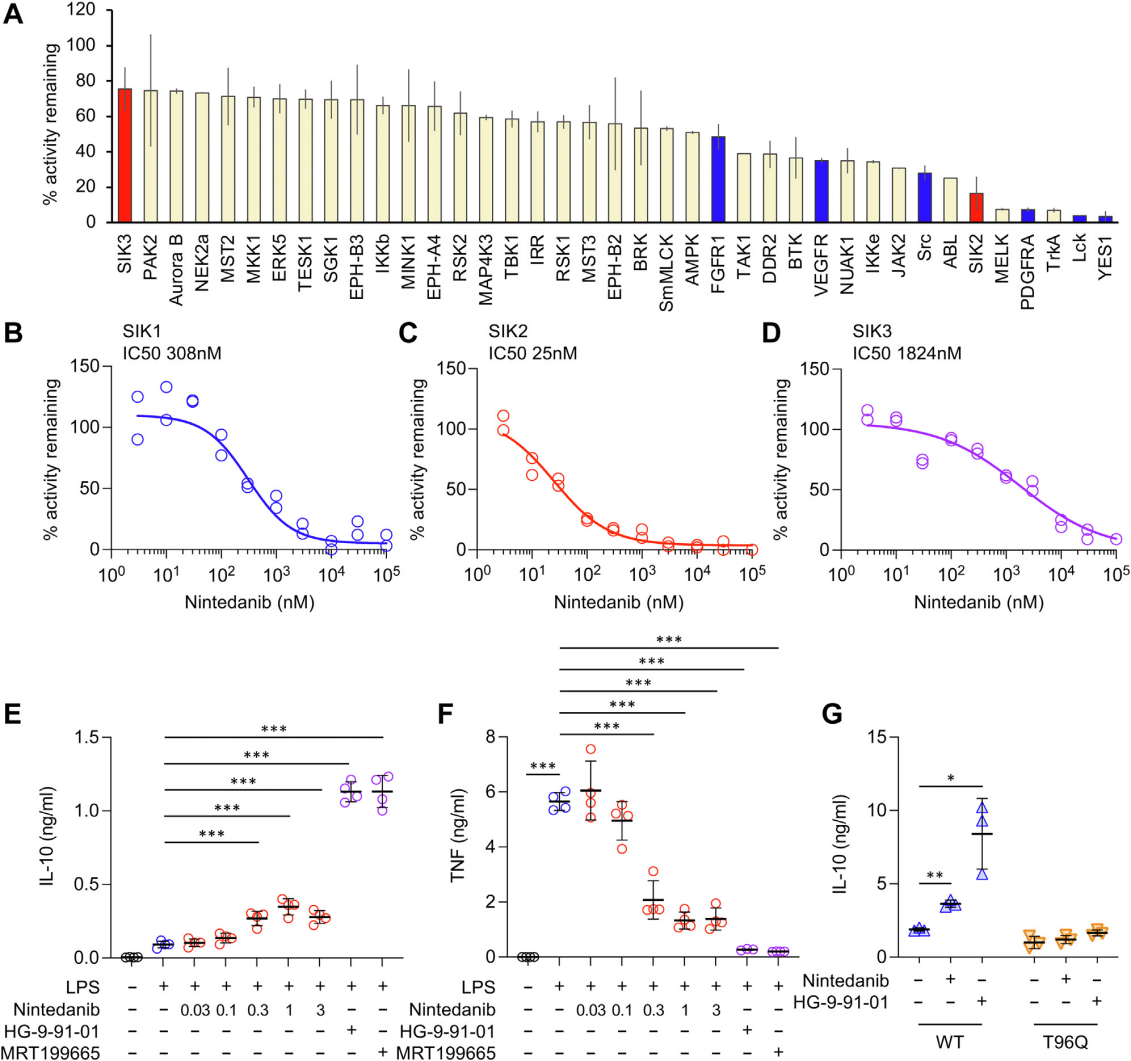
**Nintedanib inhibits SIK2 *in vitro* and in cells.***A*, nintedanib was screened against a panel of 140 kinases at 0.1 μM. The percent inhibition for the top 39 kinases is shown. Results for all 140 kinases are given in Table S1. *B*–*D*, nintedanib was tested for its ability to inhibit SIK1 (*B*), SIK2 (*C*), and SIK3 (*D*) *in vitro* at a range of concentrations from 3 nM to 100 μM. IC_50_ values were calculated as described in the Experimental procedures section. *E*, where indicated, BMDMs were incubated with 0.03, 0.1, 0.3, 1, or 3 μM nintedanib, 0.5 μM HG-9-91-01, or 1 μM MRT199665 for 1 h and then stimulated for 2 h with 100 ng/ml LPS. The levels of IL-10 secreted into the media were then determined. Graphs show the mean and standard deviation of four mice with *symbols* indicating the values for individual mice. One-way ANOVA (*F* = 259.3, *p* < 0.001) with Dunnett’s post hoc testing was used to determine significance relative to stimulation with LPS alone. A *p* < 0.001 is indicated by ∗∗∗. *F*, as (*E*) but levels of TNF were measured following 6 h of LPS stimulation (one-way ANOVA, *F* = 81.3, *p* < 0.001). *G*, RAW264.7 cells expressing either wildtype or T96Q SIK2 were treated with either DMSO, 3 μM nintedanib, or 0.5 μM HG-9-91-01 and then stimulated with 50 ng/ml LPS for 4 h. IL-10 levels were then measured in the culture supernatant. Graphs show mean and standard deviation. Inhibitor-treated conditions were compared with the DMSO control using unpaired *t* tests with Welch’s correction. *p* < 0.05 and *p* < 0.01 are indicated by ∗ and ∗∗, respectively. BMDM, bone marrow–derived macrophage; DMSO, dimethyl sulfoxide; IL, interleukin; LPS, lipopolysaccharide; SIK, salt-inducible kinase; TNF, tumor necrosis factor.

Nintedanib was developed as a tyrosine kinase inhibitor and has been approved for the treatment of IPF (38, 39). Nintedanib has been suggested to be effective in IPF because it inhibits platelet-derived growth factor receptor (PDGFR), vascular endothelial growth factor receptor (VEGFR), and fibroblast growth factor receptor (FGFR), but it also inhibits some nonreceptor tyrosine kinases, such as Src, Yes, and Lck (41). Like these tyrosine kinases, members of the SIK subfamily of protein kinases contain an amino acid with a small side chain (threonine) at the gatekeeper site of the ATP-binding pocket, and for this reason, several tyrosine kinase inhibitors are also potent SIK inhibitors (34). We therefore investigated whether nintedanib also inhibits SIKs. Initially, nintedanib was profiled at 0.1 and 1 μM against a panel of 140 kinases, which included SIK2 and SIK3 (Table S1). Analysis of the tyrosine kinases in the panel showed that, as expected, nintedanib inhibited PDGFR, VEGFR, and FGFR as well as the Src family kinases Src, Yes, and Lck (Fig. 1*A* and Table S1). Nintedanib also inhibited SIK2 to a similar degree as its known tyrosine kinase targets. To confirm this result, the IC_50_ values for inhibition of SIK1, SIK2, and SIK3 were determined (Fig. 1, *B*–*D*). Nintedanib inhibited SIK2 with an IC_50_ of 25 nM (95% confidence interval [CI]: 19–37 nM). This compares favorably with its established targets for which the published IC_50_ values range from 13 to 108 nM (41). In contrast, nintedanib was less effective against SIK1 and SIK3 with IC_50_ values of 308 nM (95% CI: 221–655 nM) for SIK1 and 1824 nM (95% CI: 732–3953 nM) for SIK3. As loss of SIK2 activity in macrophages is known to increase IL-10 production in response to lipopolysaccharide (LPS) (33), we next tested if nintedanib could also increase IL-10 production. Nintedanib increased IL-10 production by approximately threefold in LPS-stimulated bone marrow–derived macrophages (BMDMs) with a maximal effect seen between 0.3 and 1 μM (Fig. 1*E*). The increase in IL-10 production was however not as great as seen with either of the pan-SIK inhibitors, HG-9-91-01 and MRT199665 (Fig. 1*E*). Genetic studies have shown that there is compensation between different SIK isoforms in macrophages, and that loss of the activity of all three SIKs is required for the maximal effect on IL-10 (33). Loss of SIK2 activity has also been reported to reduce LPS-stimulated TNF production (33). Consistent with the effects of nintedanib on IL-10, nintedanib also reduced TNF production by macrophages with the maximal effect reached between 0.3 and 1 μM and an EC_50_ of 0.15 μM (Fig. 1*F*). For comparison, published EC_50_ values for the inhibition of proliferation of VEGFR-, PDGFR-, or FGFR-dependent cell lines range from 0.007 to 0.3 μM (41, 42). To confirm that nintedanib was affecting IL-10 and TNF production primarily *via* the inhibition of SIK2, the effects of 3 μM nintedanib treatment were compared in wildtype macrophages and macrophages from knockin mice in which SIK2 was replaced by a kinase-inactive mutant. These experiments showed that nintedanib did not affect IL-10 or TNF secretion in SIK2 knockin macrophages and that the levels of IL-10 and TNF secretion were similar in the nintedanib-treated macrophages from wildtype mice and non-inhibitor treated macrophages from SIK2 knockin mice following LPS stimulation (Fig. S1). For some kinases, mutation of the gatekeeper site in the ATP pocket to a larger residue is able to block the binding of some ATP competitive inhibitors without reducing kinase activity. An example of this is mutation of Thr96 to glutamine in SIK2, which has previously been found to prevent the binding of HG-9-91-01 (32). In RAW264.7 cells expressing a wildtype version of SIK2, both nintedanib and HG-9-91-01 were able to increase IL-10 secretion following LPS stimulation relative to cells in the absence of inhibitor, similar to what was observed in BMDMs. In contrast, in cells expressing a T96Q mutation of SIK2, IL-10 production was not promoted by either nintedanib or HG-9-91-01 (Fig. 1*G*). Together, these results suggest that nintedanib selectively inhibits SIK2 in macrophages and not SIK1 and SIK3 when used at 3 μM.

### Loss of SIK2 activity does not prevent the expression of IL-4-induced markers in BMDMs

**Figure 2:**
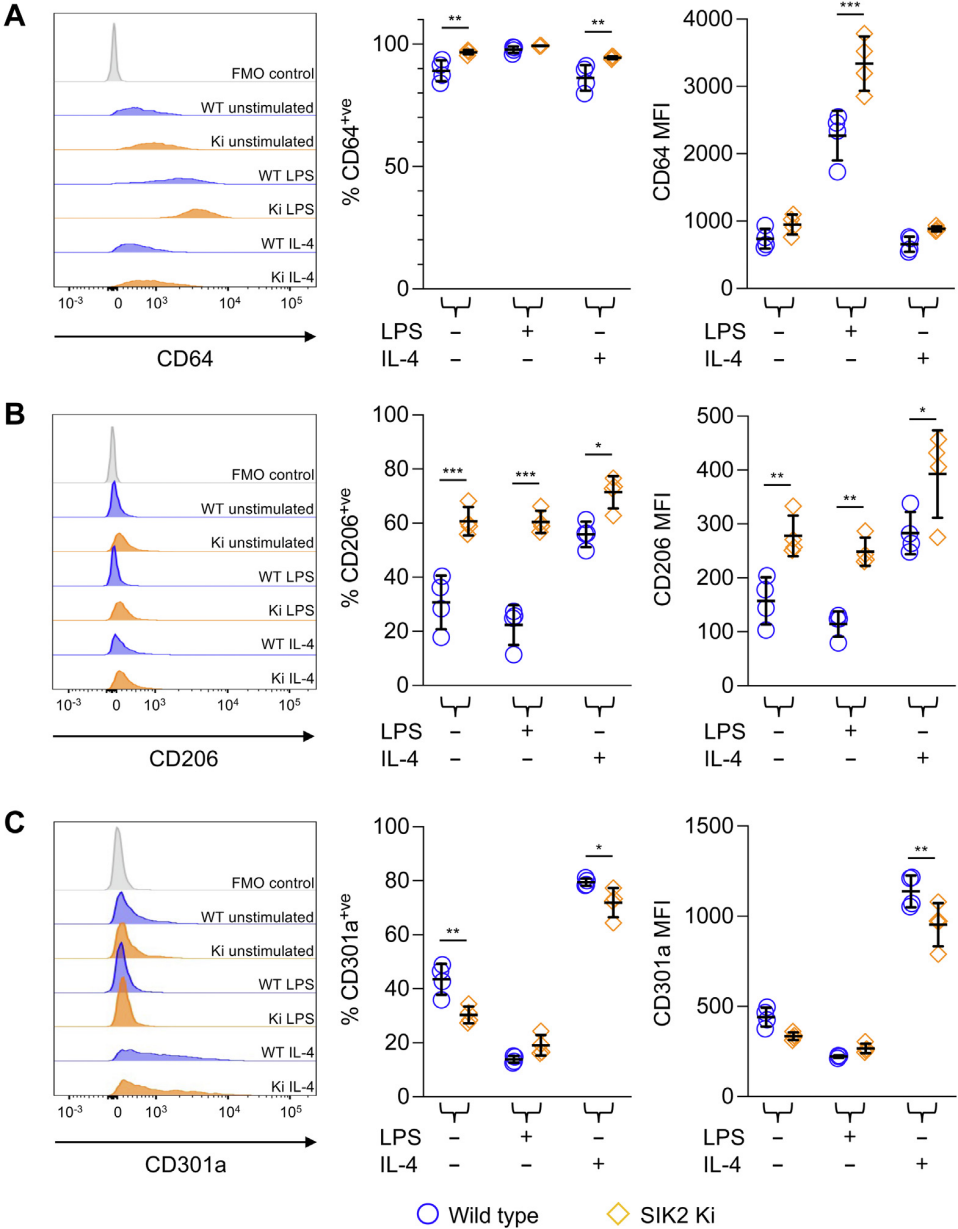
**Effect of SIK2 knockin (Ki) on macrophage marker expression.** BMDMs were generated from wildtype (*blue circles*) or SIK2 Ki (*orange diamonds*) mice. Cells were stimulated for 24 h with either 100 ng/ml LPS or 10 ng/ml IL-4. Cells were then stained for F4/80, CD301a, and either CD206 or CD64. Live F4/80^+ve^ cells were gated, and the mean fluorescence intensity (MFI) for CD64 (*A*), CD206 (*B*), or CD301a (*C*) was determined. Graphs show mean and standard deviation with symbols representing individual mice. A *p* value of less than 0.05 is indicated by ∗, less than 0.01 by ∗∗, and less than 0.001 by ∗∗∗ (two-way ANOVA followed by Sidak’s post hoc testing; *F* and *p* values for the ANOVAs are shown in Table S3). BMDM, bone marrow–derived macrophage; IL, interleukin; LPS, lipopolysaccharide; SIK, salt-inducible kinase.

Given that lung fibrosis in mice has been linked to increased polarization of macrophages to an IL-4-induced “M2a” phenotype, the effect of the SIK2 kinase-inactive knockin was tested on IL-4 and LPS-induced markers on macrophages. LPS induces the expression of CD64, which has been used as a marker of inflammatory M1 macrophages (43). Analysis of CD64 expression by flow cytometry showed that CD64 was expressed on BMDMs prior to stimulation and was upregulated following LPS stimulation (Fig. 2*A*). While SIK2 knockin decreases proinflammatory cytokine production in response to LPS, unexpectedly, it increased CD64 expression relative to wildtype macrophages following LPS stimulation (Fig. 2*A*). IL-4 is known to induce the expression of CD206 and CD301a in macrophages (44, 45). CD206 expression was low in wildtype BMDMs but was increased following IL-4 stimulation (Fig. 2*B*). SIK2 knockin resulted in a higher expression of CD206 relative to wildtype cells, both in unstimulated cells and following IL-4 stimulation (Fig. 2*B*). CD301a expression was also increased by IL-4 treatment in wildtype BMDMs (Fig. 2*C*). However, in contrast to CD206 expression, SIK2 knockin resulted in lower CD301a expression compared with wildtype cells, both before and after IL-4 stimulation (Fig. 2*C*).

**Figure 3:**
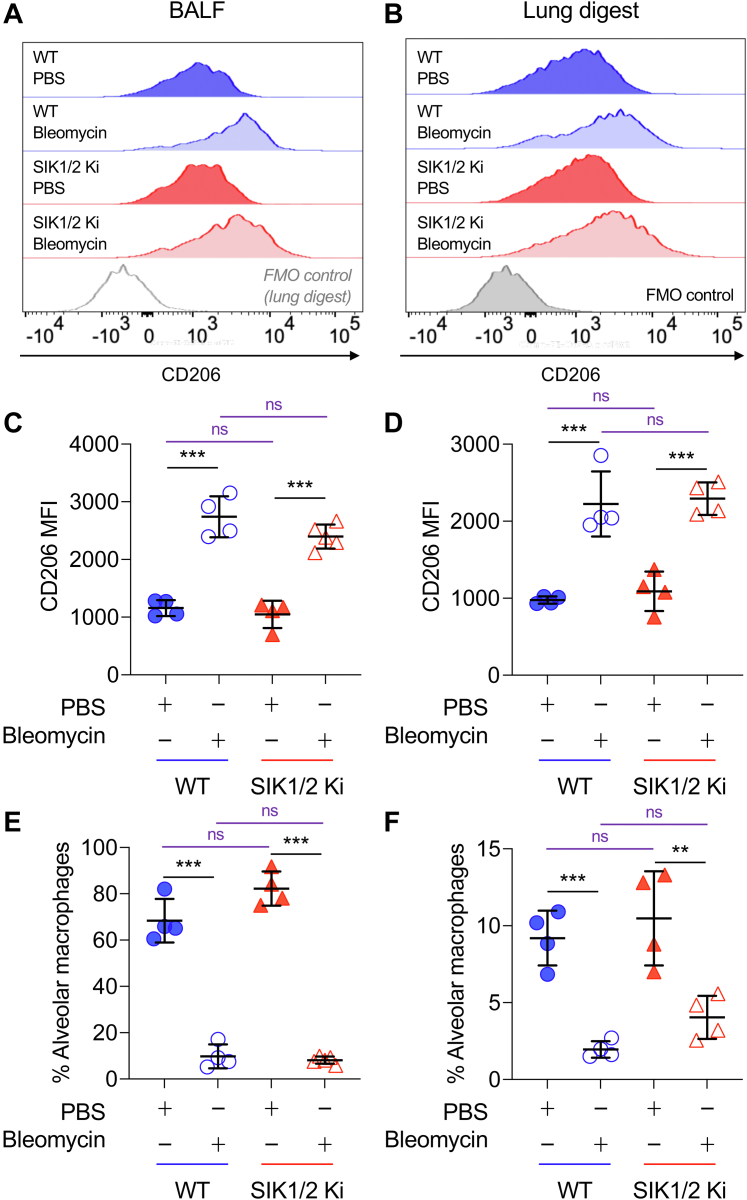
**Expression of CD206 in wildtype and SIK1/2 knockin (Ki) alveolar macrophages.** Wildtype and SIK1/2 Ki mice were given an oropharyngeal dose of 1.5 mg/kg bleomycin or an equivalent volume of PBS. Mice were culled on day 7, and cells in the BALF (*A*, *C*, and *E*) and lung digests (*B*, *D*, and *F*) were stained with CD45, CD11c, Siglec F, CD206, F4/80, Ly6G, and CD11b and analyzed by flow cytometry. Representative histograms for CD206 expression in CD45^+ve^/Siglec F^+ve^/CD11c^+ve^ alveolar macrophages are shown in (*A* and *B*), and mean fluorescent intensity for CD206 in alveolar macrophages is shown in (*C* and *D*). The number of alveolar macrophages as a percentage of live cells is shown in (*E* and *F*). Four to 5 mice were analyzed per group, and graphs show mean and standard deviation with individual mice shown by *symbols*. Data were analyzed by two-way ANOVA with Sidak’s post hoc testing to compare between PBS and bleomycin (*black*) or wildtype and SIK1/2 Ki genotypes (*purple*). *p* < 0.05, 0.01, and 0.001 is indicated by ∗, ∗∗, and ∗∗∗, respectively; ns indicates *p* > 0.05. *F* and *p* values for the ANOVAs are shown in Table S4. BALF, bronchoalveolar lavage fluid; SIK, salt-inducible kinase.

As these results might suggest a change in macrophage polarization *in vivo*, the expression of CD206 was examined in alveolar macrophages. CD206 expression has been reported in alveolar macrophages and found to be increased following short-term bleomycin treatment (46). In line with this, CD206 was detected on CD45^+ve^/Siglec F^+ve^/CD11c^+ve^ alveolar macrophages present in both the bronchoalveolar lavage fluid (BALF) (Fig. 3*A*) and in digests of lung tissue (Fig. 3*B*). These levels were increased 7 days post-treatment with bleomycin (Fig. 3, *C* and *D*) although the numbers of CD45^+ve^/Siglec F^+ve^/CD11c^+ve^ macrophages that could be detected in either the BALF or in lung digests decreased following bleomycin treatment (Fig. 3, *E* and *F*). In contrast to BMDMs, the level of CD206 in alveolar macrophages was similar between wildtype and SIK1/2 knockin cells. The loss of SIK1 and SIK2 activity also did not affect the decrease in alveolar macrophage numbers seen following bleomycin treatment. While CD206 was detected on alveolar macrophages, it was only present at very low levels on CD45^+ve^/CD11c^−ve/^CD11b^+ve^/Ly6G^−ve^ or CD45^+ve^/CD11c^−ve/^CD11b^+ve^/Ly6G^+ve^ myeloid cells in the lungs of mice, and these levels were not affected by SIK1/2 knockin (Fig. S2).

### SIK2 regulates bleomycin-induced lung fibrosis

**Figure 4:**
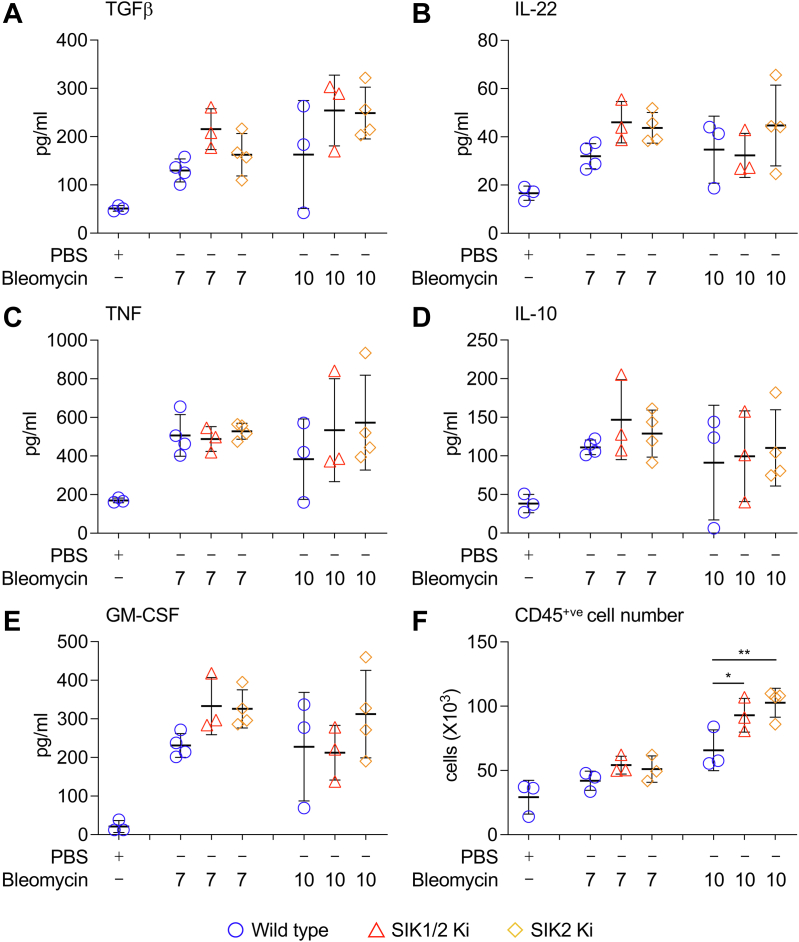
**Analysis of initial bleomycin-induced inflammation in wildtype and SIK1/2 knockin (Ki) mice.** Wildtype, SIK2, or SIK1/2 Ki mice were given an oropharyngeal dose of 2 mg/kg bleomycin or a PBS control. Mice were sacrificed on day 7 or 10 after bleomycin administration, and the levels of TGFβ (*A*), IL-22 (*B*), TNF (*C*), IL-10 (*D*), and GM-CSF (*E*) in the BALF were determined using a bead-based multiplex ELISA as described in the Experimental procedures section. The number of CD45^+ve^ immune cells in the BALF (*F*) was determined by flow cytometry. Graphs show mean and standard deviation with *symbols* representing individual mice. ∗ and ∗∗ indicate *p* < 0.05 and 0.01, respectively (two-way ANOVA followed by Tukey’s post hoc testing). *F* and *p* values for the ANOVAs are shown in Table S5. BALF, bronchoalveolar lavage fluid; GM-CSF, granulocyte–macrophage colony-stimulating factor; IL, interleukin; SIK, salt-inducible kinase; TGFβ, transforming growth factor beta; TNF, tumor necrosis factor.

Bleomycin administration in the lungs of mice results in an initial inflammatory phase lasting 7 to 10 days that then resolves into a fibrotic phase. To examine a role for SIK2 in this initial inflammatory phase, mice were sacrificed at 7 and 10 days following bleomycin treatment, and the presence of immune cells and cytokines in the BALF was determined. TGFβ has been implicated in the development of bleomycin-induced fibrosis (47, 48). Levels of TGFβ were increased in the BALF following bleomycin treatment; however, no significant differences (*p* > 0.05, two-way ANOVA) were observed between wildtype, SIK2, and SIK1/2 knockin mice (Fig. 4*A*). IL-22 is another cytokine induced by bleomycin treatment, and knockout of IL-22 or its neutralization using a monoclonal IL-22 antibody reduced bleomycin-induced lung injury (49). Similar to TGFβ, induction of IL-22 was comparable between wildtype, SIK2, and SIK1/2 knockin mice (Fig. 4*B*). TNF is also increased following bleomycin treatment, and a role for TNF in promoting the resolution of inflammation by stimulating apoptosis in immune cells has been proposed in the bleomycin model (50, 51). The induction of TNF was however not affected by the loss of SIK2 or SIK1/2 activity (Fig. 4*C*). The levels of granulocyte–macrophage colony-stimulating factor and IL-10, cytokines implicated in limiting inflammation in the bleomycin model (52, 53, 54), were also increased following bleomycin treatment, but again, no significant differences were observed between wildtype, SIK2, or SIK1/2 knockin mice (Fig. 4, *D* and *E*). In addition to cytokines, several chemokines, including CCL2 and CXCL2, have been reported to be increased in mice following the administration of bleomycin and are required for the induction of bleomycin-induced pathology (14, 55, 56). Consistent with this, CCL2 and CXCL2 were found to be increased in the BALF of bleomycin-treated mice, but again, this was not affected by SIK2 or SIK1/2 knockin (Fig. S3, *A* and *B*). Bleomycin was also able to increase the number of CD45-positive immune cells present in the BALF (Fig. 4*F*). Both the SIK2 and SIK1/2 knockin mice showed a slight increase in immune cell recruitment 10 days after bleomycin treatment (Fig. 4*F*). While in naïve mice, the cells in the BALF were mostly alveolar macrophages, these decreased following bleomycin treatment, whereas a number of other immune cell types, including neutrophils, eosinophils, B cells, and both CD4 and CD8 T cells, were recruited to the BALF (Fig. S3, *C*–*H*). The percentages of these different cell types were however similar between the bleomycin-treated wildtype, SIK2, and SIK1/2 knockin mice (Fig. S3, *C*–*H*).

**Figure 5:**
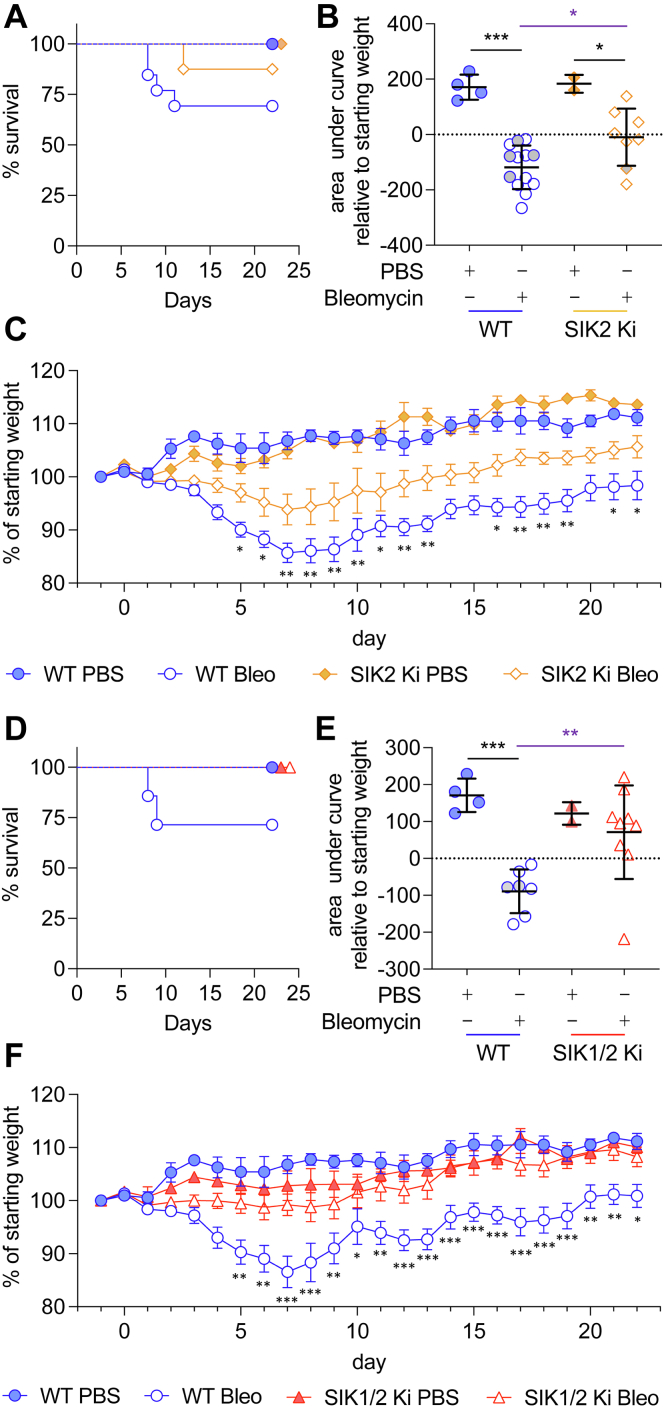
**Bleomycin (bleo)-induced weight change in wildtype, SIK2, and SIK1/2 knockin (Ki) mice.** Mice were given an oropharyngeal dose of 2 mg/kg of bleo or an equivalent volume of PBS. Weight was monitored daily for 22 days. Mice that lost more than 20% of their body weight without showing signs of improvement within 3 days were considered to have reached the humane end point and were sacrificed at that point. *A*–*C*, wildtype and SIK2 Ki mice were treated with either PBS or bleo. The percentage survival is shown in (*A*). The area above or below the curve relative to the starting weight is shown in (*B*). Positive values indicate increases, and negative values indicate decreases in weight. Average and standard deviation are shown, with *symbols* representing individual mice. *Gray symbols* indicate mice that did not reach the 22 day end point. The percentage weight change on each day relative to the weight 1 day before bleo treatment is shown in (*C*). Data show average values for each group, and error bars represent SEM, with initial group sizes of four and two for the PBS-treated wildtype and SIK2 Ki mice and 13 (six males and seven females) and 8 (four males and four females) for the bleomycin-treated wildtype and SIK2 Ki mice, respectively. *D*–*F*, as (*A*–*C*), except female wildtype and SIK1/2 double Ki mice were analyzed, with survival shown in (*D*), area under the curve in (*E*), and weight changes in (*F*). Initial group sizes were four for wildtype PBS-treated mice, two for SIK1/2 Ki PBS-treated mice, seven for wildtype bleomycin-treated mice, and nine for the SIK1/2 Ki bleomycin-treated group. For (*B* and *E*), *p* < 0.05 and *p* < 0.001 are shown by ∗ and ∗∗∗ (two-way ANOVA and Tukey’s post hoc testing). For (*C* and *F*), data were analyzed using a mixed-effects model and Tukey’s post hoc testing. For comparisons on individual days between wildtype and SIK2 or SIK1/2 Ki bleomycin-treated mice, ∗ indicates *p* < 0.05, ∗∗ indicates *p* < 0.01, and ∗∗∗ indicates *p* < 0.001. Full statistical analysis is shown in Table S6. SIK, salt-inducible kinase.

**Figure 6:**
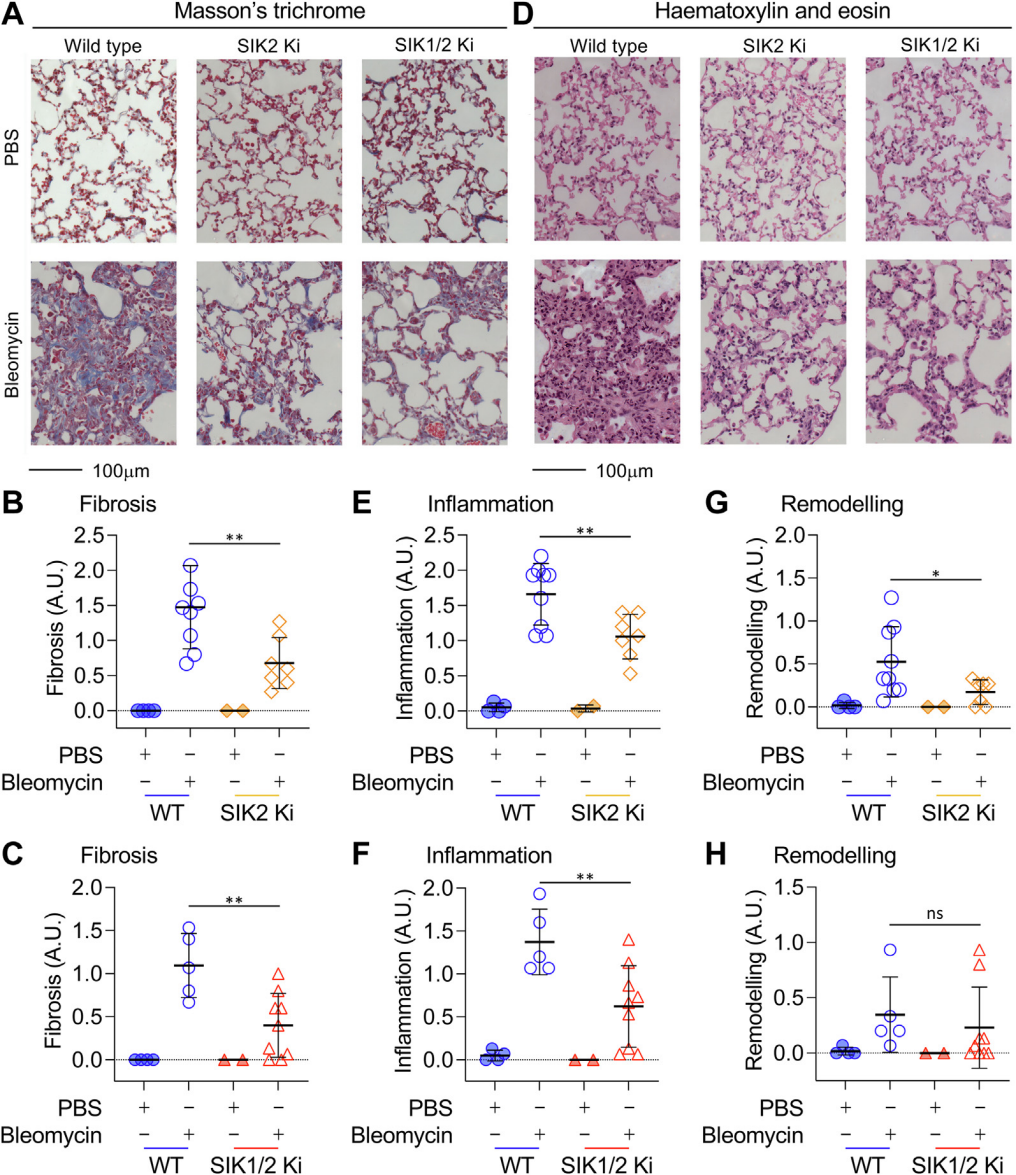
**Analysis of bleomycin-induced lung pathology in wildtype, SIK2, and SIK1/2 knockin mice.** Mice were given an oropharyngeal dose of 2 mg/kg of bleomycin or a PBS control. On day 22, mice were sacrificed, and lungs were fixed and stained with either Masson's trichrome or H&E as described in the Experimental procedures section. The data are derived from the surviving mice used in the experiments described for Figure 5. Representative images for Masson's trichrome staining are shown in (*A*) and scoring for fibrosis in (*B* and *C*). Representative images for H&E staining are shown in (*D*) with scoring for inflammation in (*E*) and (*F*) and type II hyperplasia in (*G* and *H*). Graphs show mean and standard deviation with *symbols* showing individual mice. *p* < 0.05 is indicated by ∗ and *p* < 0.01 by ∗∗ (two-way ANOVA followed by Sidak’s post hoc testing, *F* and *p* values for the ANOVAs are shown in Table S7). SIK, salt-inducible kinase.

As SIK2 did not appear to affect the initial inflammatory phase of bleomycin-induced lung inflammation, we next examined its potential effects on fibrosis using a 22 day model. Following administration of bleomycin, wildtype mice lost weight with peak weight loss occurring 7 to 10 days after the administration of bleomycin, after which mice started to regain weight (Fig. 5). Mice that lost more than 20% of their body weight without showing signs of improvement within 3 days were considered to have reached the humane end point and were sacrificed. Four of the 13 bleomycin-treated wildtype animals reached this point between day 7 and 11 of the protocol (Fig. 5*A*). SIK2 knockin mice showed a lower dropout rate, with one of eight mice reaching the humane end point before the end of the experiment (Fig. 5*A*). To estimate weight loss over the whole experiment, the area under the curve was calculated for each mouse relative to its starting weight. Using this measure, SIK2 single knockin mice lost significantly less weight (*p* < 0.05) than wildtype mice following bleomycin treatment (Fig. 5*B*). Consistent with this, SIK2 bleomycin-treated mice showed a lower peak weight loss relative to bleomycin-treated wildtype animals (Fig. 5*C*). Similar results were also obtained when SIK1/2 double knockin mice were analyzed, with the SIK1/2 knockin animals showing increased survival (Fig. 5*D*) as well as decreased weight loss (Fig. 5, *E* and *F*). The extent of fibrosis at day 22 was assessed by histological scoring of Masson’s trichrome-stained lung sections. As expected, extensive collagen deposition throughout the alveolar bed (stained *blue* in Fig. 6*A*) was apparent in the lungs from bleomycin-treated wildtype mice. Both the SIK2 (Fig. 6, *A* and *B*) and SIK1/2 (Fig. 6, *A* and *C*) knockin mice showed reduced levels of collagen deposition compared with the wildtype animals.

Lung sections were also stained with H&E to assess inflammation and type II pneumocyte hypertrophy/hyperplasia (Fig. 6*D*). In addition to the reduction in fibrotic score relative to wildtype animals, at day 22, the lungs from bleomycin-treated SIK2 and SIK1/2 knockin mice showed lower inflammation scores than bleomycin-treated wildtype mice (Fig. 6, *E* and *F*). Type II pneumocyte hypertrophy/hyperplasia was also decreased in SIK2 knockin mice relative to wildtype (Fig. 6*G*); however, this change was not significant in SIK1/2 knockin mice (Fig. 6*H*, *p* > 0.05).

**Figure 7:**
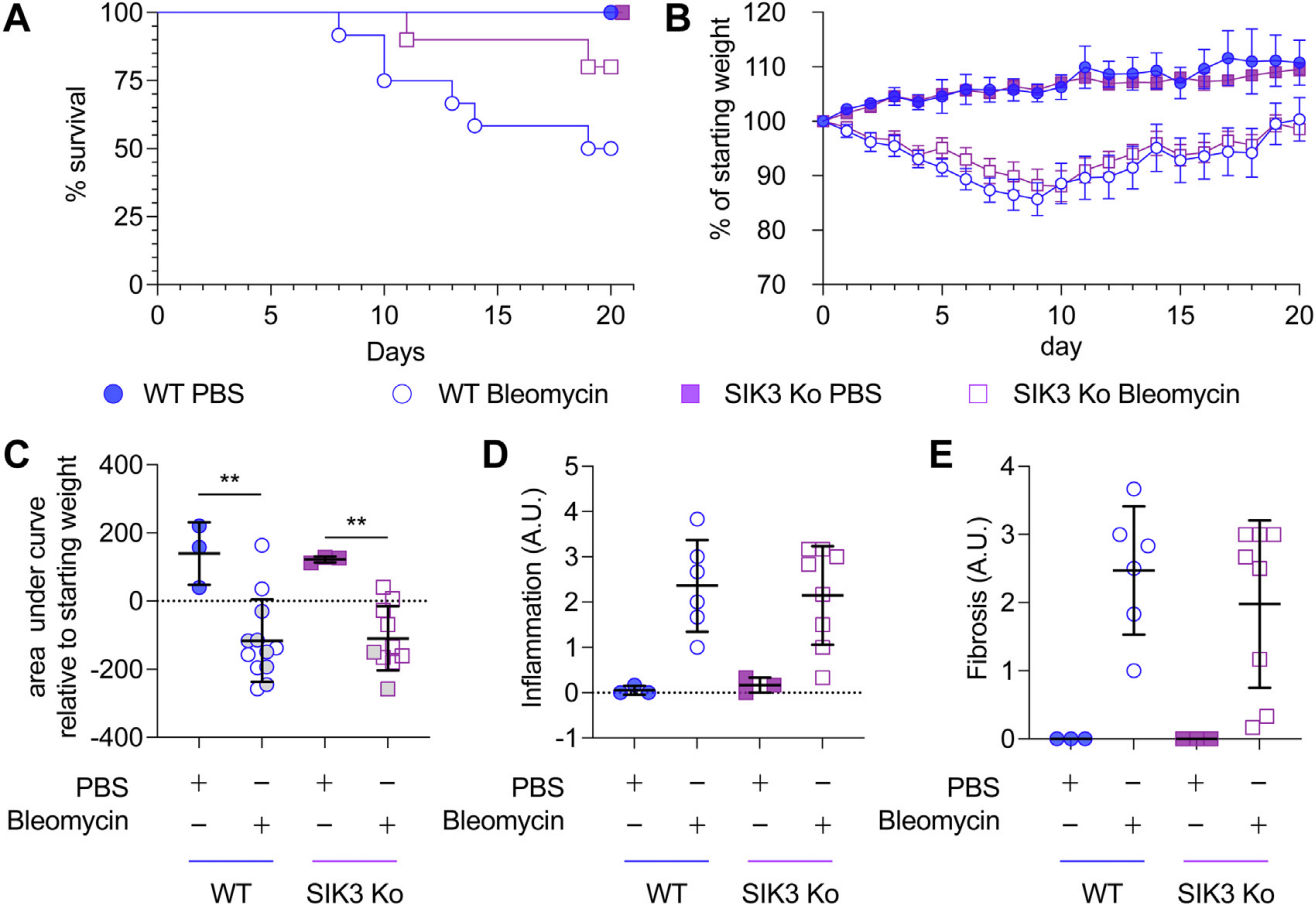
**Bleomycin-induced fibrosis in wildtype and immune cell–specific SIK3 knockout (Ko) mice.** Wildtype Vav-iCre^+ve^ or immune cell–specific SIK3^fl/fl^/Vav-iCre^+ve^ Ko mice were given an oropharyngeal dose of 1.5 mg/kg of bleomycin or a PBS control. Weight was monitored daily for 20 days. Mice that lost more than 20% of their body weight without showing signs of improvement in 3 days were considered to have reached the humane end point and sacrificed. The percentage survival is shown in (*A*). The average percentage weight change relative to the weight on day 0 before bleomycin treatment is shown in (*B*). Error bars represent SEM, with initial group sizes of three for the PBS-treated wildtype and immune cell–specific SIK3 knockout mice and 12 (six males/six females) and 10 (six males/four females) for the bleomycin-treated wildtype and immune cell–specific SIK3 knockout mice, respectively. Mixed-effects model analysis showed no significant difference between wildtype and SIK3 knockout bleomycin-treated mice (*p* > 0.05). The area above or below the curve relative to the starting weight is shown in (*C*). Positive values indicate increases in weight, and negative values indicate decreases in weight. Average and standard deviation are shown with *symbols* representing individual mice. *Gray symbols* indicate mice that did not reach the 20 day end point. *p* < 0.01 is indicated by ∗∗ (two-way ANOVA and Sidak’s post hoc testing). On day 20, mice were sacrificed, and lungs were fixed and stained with either H&E or Masson's trichrome as described in the Experimental procedures section. Scoring for fibrosis is shown in (*E*), and scoring for inflammation is shown in (*D*). Graphs show mean and standard deviation with *symbols* showing individual mice. Two-way ANOVA testing did not identify significant (*p* > 0.05) differences between the wildtype and immune cell–specific SIK3 knockout mice. *F* and *p* values for the mixed-model analysis and ANOVAs are shown in Table S8. SIK, salt-inducible kinase.

As well as SIK2, immune cells also express SIK3. Both SIK3 knockout and SIK3 kinase inactive knockin mice exhibit increased postnatal mortality and growth retardation (33, 57), making them poorly suited for the analysis of SIK3 in lung fibrosis. To address the role of SIK3 in immune cells in bleomycin-induced fibrosis, we generated a hemopoietic cell–specific knockout of SIK3 by crossing floxed SIK3 mice to a Vav-iCre transgene (58). Following bleomycin treatment, survival was higher in the SIK3 knockout (8 of 10) relative to the wildtype group (6 of 12, Fig. 7*A*) although this did not reach significance for the group size analyzed (*p* = 0.133, log-rank Mantel–Cox test). Despite this, no difference in weight loss was observed between wildtype and the immune cell–specific SIK3 knockout mice, either when individual time points (Fig. 7*B*) or the area under the curve (Fig. 7*C*) were compared. Histological analysis of the lungs at day 20 did not show significant differences between the wildtype and SIK3 knockout in the extent of fibrosis or inflammation (Fig. 7, *D* and *E*).

## Discussion

IPF is a fatal condition that remains difficult to treat; however, the approval of nintedanib in 2014 was a significant advance in the management of this condition (39). Since then, nintedanib has also shown positive results in phase 3 trials for interstitial lung disease associated with systemic sclerosis (59, 60) and progressive fibrosing interstitial lung diseases (61, 62) and has been approved for these conditions.

Nintedanib is a tyrosine kinase inhibitor, and its success in IPF has been attributed to the inhibition of several tyrosine kinase receptors, including the VEGFR, PDGFR, and FGFR (36, 38, 41). It does however inhibit several other kinases that, like its receptor tyrosine kinase targets, contain a small amino acid at the gatekeeper site that is required for access of inhibitors to the ATP-binding pocket. The possibility that off-target effects of nintedanib on one or more of these kinases contributes to its effectiveness in IPF cannot be excluded. We show here that nintedanib inhibits SIK2 with similar potency to its known tyrosine kinase targets both *in vitro* and in cells. Supporting a role for SIK2, a recent study has shown that a compound developed as an SIK inhibitor, ARN-3236, is able to attenuate bleomycin-induced lung fibrosis (63). ARN-3236 has selectivity for SIK2 over SIK1 and SIK3 with *in vitro* IC_50_ values reported as <1, 21.6, and 6.6 nM, respectively (64). SIK inhibitors frequently have off-target activity against tyrosine kinases, and in this respect, ARN-3236 is no different; it is a potent inhibitor of Lck, a kinase critical for T-cell receptor function, and also has activity against the VEGFR (64). Its activity against the PDGFR, another key target of nintedanib in fibrosis, was not determined in this study. While the ability of both nintedanib and ARN-3236 to inhibit SIK2 is suggestive of a role for this kinase in fibrosis, their ability to inhibit selected tyrosine kinases means further evidence is required to support a role for SIK2 in this process. We show that SIK2 kinase-inactive knockin mice are protected in a bleomycin-induced lung fibrosis model. While nintedanib was an effective SIK2 inhibitor, it was less effective at inhibiting SIK1 and SIK3. Interestingly, this was mirrored by the effects of SIK2 kinase-inactive knockin mutations in bleomycin-induced lung fibrosis. Inactivation of SIK1 in addition to SIK2 resulted in similar levels of protection compared with SIK2 inactivation alone, suggesting that SIK2 is the more important isoform. While we were unable to use SIK3 kinase–inactive knockin mice, because of adverse developmental phenotypes in these mice, we were able to examine an immune cell–specific SIK3 knockout and found that this did not effectively protect against bleomycin-induced fibrosis. It is however possible that compensation between SIK2 and SIK3 may occur in fibrosis. It would therefore have been of interest to examine SIK2/3 double knockin mice; however, this was not possible as loss of both SIK2 and SIK3 activity results in embryonic lethality (33). Furthermore, although mice with a double knockout of both SIK2 and SIK3 in the immune system are viable, they exhibit a failure of thymic T-cell development and very low numbers of peripheral T cells (65), which would make interpreting the results of bleomycin-induced fibrosis problematic.

The molecular mechanism by which SIK2 regulates fibrosis is unclear. TGFβ has been proposed to play a central role in bleomycin-induced fibrosis; however, the induction of TGFβ was not affected by loss of SIK2 activity. Further work will therefore be required to determine the targets of SIK2 during the development of lung fibrosis. Bleomycin-induced fibrosis has been associated with an increase in expression of markers of IL-4-induced activation in macrophages (17, 18, 19, 20, 21). While this may be important in fibrosis, it is not straightforward to show a direct link between macrophage polarization and fibrosis. Transcriptomic profiling has shown that macrophages can adopt multiple heterogenous states *ex vivo* depending on the signaling inputs they receive (66). With relation to bleomycin-induced fibrosis, transcriptional analysis of monocyte-derived and tissue resident alveolar macrophages show they both upregulate a subset of M1- and M2-associated genes, with no clear indication of an overall change in the M1/M2 ratio (16). While loss of SIK2 activity decreases the production of inflammatory cytokines in macrophages (33), it had contrasting effects on markers for IL-4 stimulation in BMDMs, with CD206 expression being elevated but CD301a expression slightly decreased (Fig. 2). Of note, in BMDMs, the effect of SIK2 inhibition on CD206 expression was reduced following IL-4 stimulation, suggesting it is dependent on the cytokine environment of the macrophages. In contrast to BMDMs, loss of SIK2 activity did not affect CD206 expression in alveolar macrophages. This could reflect the different cytokine environments seen between cultured macrophages and the cells in the lung or the different origins of the cells; BMDMs are derived *ex vivo* from bone marrow precursors stimulated with M-CSF, whereas alveolar macrophages in naïve mice are seeded in embryonic and early postnatal development and require granulocyte–macrophage colony-stimulating factor (67, 68).

Both CD206 and CD301a are members of the C-type lectin receptor family. While increases in CD206 expression on macrophages following bleomycin treatment is a useful marker for macrophage activation, it is less clear whether it plays an active role in the development of pathology. A recent report suggests that peptides targeting CD206 are protective in bleomycin-induced fibrosis by promoting cell death in CD206^+ve^ macrophages (69). Nanoparticles targeting TGFβ siRNA to CD206^+ve^ macrophages can also reduce bleomycin-induced fibrosis (15). While these findings support a role for CD206^+ve^ macrophages, it does not address if CD206 is directly important in the response to bleomycin or if it only acts as a marker for alveolar macrophages that allows them to be targeted *in vivo*. The role of CD301a has not been addressed in bleomycin-induced fibrosis, although knockout of CD301a has been found to reduce tissue remodeling in antigen-induced granuloma formation (70). While SIKs are able to inhibit the inflammatory functions of macrophages in culture, it is not clear if this is sufficient to explain their roles in lung fibrosis. Loss of SIK2 activity did not reduce the initial inflammatory phase following bleomycin treatment and did not promote IL-10 production in this model *in vivo*. This contrasts with multiple studies showing that SIK inhibition in isolated macrophages promotes IL-10 production *via* promoting CREB activity (31, 32, 33, 64). This could be due to the bleomycin-induced IL-10 in the lung being derived from a different cellular source, such as T cells or B cells where CREB does not seem to be critical for its production (29). It should also be noted that for SIK inhibition in cultured macrophages to have its maximal effect on either IL-10 or the phosphorylation of the CREB coactivator CRTC3, all three SIK isoforms have to be inhibited. In contrast to this, SIK2 appears to be the predominant isoform regulating lung fibrosis, although a contribution from SIK3 cannot be ruled out. Together, this suggests that during bleomycin-induced lung fibrosis, the main effects of SIK2 inhibition may not be due to its anti-inflammatory role in macrophages, and that it could potentially be acting *via* a novel substrate. It should be noted that SIK2 appears to be ubiquitously expressed (33), and as the knockin mice used were not conditional, it is not possible to say in what cell types SIK2 functions during the development of fibrosis. Further work will be needed to resolve these issues.

Together, the data presented here identify SIK2 as a target of a clinically approved antifibrotic drug and show that the SIK2 kinase-inactive knockin mouse is protected in a bleomycin-induced mouse model for lung fibrosis. While the efficacy of nintedanib was originally proposed to be due to its ability to inhibit tyrosine kinases, it is possible that at least some of its effects in the clinic are due to its ability to inhibit SIK2.

## Experimental procedures

### Animals

The generation of SIK1 and SIK2 kinase-inactive knockin mice has been described previously (33). A conditional SIK3 line was obtained from IMPC and crossed to a Vav-iCre transgenic line to generate mice lacking SIK3 in the immune system (58, 71). All animals were maintained on a C57Bl6/J background using mice from Charles River Laboratories. Mice were kept under specific pathogen-free conditions in individually ventilated cages and given free access to food (RM3 irradiated pelleted diet supplied by Special Diet Services) and water. Animal rooms were maintained at a temperature of 21 °C and a humidity of 45 to 55% and 12/12 h light cycle. All animal works were carried out under a UK Home Office license and subject to approval by the University of Dundee’s Welfare and Ethical Use of Animals Committee. Experiments were planned in accordance with the ARRIVE guidelines.

### Kinase inhibitors

Nintedanib was purchased from Tocris Bioscience. The SIK inhibitors HG-9-91-01 and MRT199665 were synthesized by Dr Natalia Shpiro and can be obtained from MRC PPU Reagents and Services (https://mrcppureagents.dundee.ac.uk/). LPS (*Escherichia coli* strain O5:B55) was obtained from Alexis Biochemicals, and recombinant murine IL-4 was from Peprotech.

### *In vitro* kinase assays

An *in vitro* selectivity screen was carried out as described (72). Human SIK1, SIK2, and SIK3 were expressed in Sf9 insect cells. To determine the IC_50_ value for SIK1, SIK2, and SIK3, nintedanib was assayed on a 10-point concentration curve from 0.003 to 100 μM. Assays were carried out in a final volume of 25.5 μl containing 50 mM Tris–HCl (pH 7.5), 0.1 mM EGTA, 0.05% (v/v) β-mercaptoethanol, 300 μM substrate peptide (ALNRTSSDSALHRRR), 10 mM magnesium acetate, and 0.05 mM [33P-γ-ATP] (50–1000 cpm/pmol) and incubated for 30 min at room temperature. Assays were started by the addition of ATP and stopped by addition of 5 μl of 0.5 M (3%) orthophosphoric acid and then harvested onto P81 Unifilter plates with a wash buffer of 50 mM orthophosphoric acid. IC_50_ values were calculated in GraphPad Prism (GraphPad Software, Inc) by nonlinear regression using the model y = ymin+ (ymax-ymin)/(1 + (x/IC_50_)), where ymax and ymin are the maximum and minimum values for percentage inhibition, respectively.

### Macrophage culture

Primary BMDMs were obtained by differentiating bone marrow from 8- to 12-week-old C57Bl6/J mice for 7 days in Dulbecco's modified Eagle's medium supplemented with 20% (v/v) L929 conditioned medium as a source of M-CSF, 2 mM l-glutamine, 10% (v/v) fetal calf serum, 1 mM pyruvate, non-essential amino acids, 10 mM Hepes, 50 μM β-mercaptoethanol, 50 U/ml penicillin, and 50 μg/ml streptomycin. Macrophages were differentiated on non–tissue culture–treated plastic, harvested, and replated at a density of 1,00,000 cells/cm^2^ on tissue culture–treated plastic in fresh media prior to stimulation on day 8. Where indicated in the figure legends, cells were incubated with inhibitors for 1 h prior to stimulation.

RAW264.7 cells with stably integrated expression cassettes for wildtype and T96Q SIK2 under the control of a tetracycline-inducible promoter have been described previously (32). Cells were cultured in Dulbecco's modified Eagle's medium supplemented with 10% (v/v) fetal bovine serum (FBS), 100 U/ml penicillin, 100 μg/ml streptomycin, 2 mM glutamine, 3 μg/ml puromycin, and 1 mg/ml G418. To induce the expression of SIK2, cells were treated with 1 μg/ml doxycycline in the absence of G418 and puromycin for 16 h before treatment with inhibitors and stimulation with LPS.

### Bleomycin-induced lung fibrosis

Mice were anesthetized with isoflurane and received a single dose of bleomycin (1.5 or 2 mg/kg; Sigma) *via* an oropharyngeal route. Mice were culled at 3, 7, 10, or 22 days following the dosing as indicated in the figure legends or when the humane end point was reached (the humane end point was reached when either mice lost more than 25% of their initial weight, mice remained for more than 3 consecutive days at a weight loss over 20% of their initial weight, when after starting to recover, they stopped recovering and stayed at a stable weight for more than 5 days with weight loss of over 10% or when body condition score reached a value of 1.5). Mice were monitored daily, with body weight measured at a fixed time every morning, starting 3 days before the dosing and continuing until the last day, and body condition was independently assessed by two people (with one scorer blind to the experimental treatment and genotype). For the duration of the experiment, mice received supplementary food in the form of rehydrated mash (same formulation as normal food) and a high fat supplement (peanut butter; Tesco brand).

Mice were culled using an overdose of anesthetic (Euthatal) followed by exsanguination *via* vena cava and blood collected in Minicollect serum collection tubes (Greiner Bio-One). For analysis of CD206 expression by flow cytometry, mice were culled *via* lethal dose of anesthetic (medetomidine HCl/ketamine HCl) followed by exsanguination. Lungs were washed three times in succession with 400 μl PBS. Supernatants were collected for BALF measurements, and cells were used for flow cytometry. The left lung was fixed in 4% (w/v) paraformaldehyde in PBS, and the right lung was divided into two, with half snap frozen in liquid nitrogen and half processed for flow cytometry.

### Histology

Fixed lung tissue was sectioned twice along the transverse axis, and the resulting three samples were processed to paraffin embedding. Serial sections were mounted and stained with Masson’s trichrome and H&E. A multiparametric scoring system (73) was developed to assess microscopic changes in the lung. Fibrosis, inflammation, and type II pneumocyte hypertrophy/hyperplasia were evaluated in 15 microscopic fields (five fields per transverse section of the left pulmonary lobe) and scored with an ordinal grading scale (0–4) developed according to published guidelines and principles for valid histopathologic scoring in research (74). Four to five score levels are considered the optimal range to maximize detection and reproducibility for semiquantitative grading applied to histopathology (74). Scoring of the histological end points was done by a veterinary pathologist blinded to genotype and treatment. The defining criteria of the scoring system are detailed in Table S2.

### Lung digestions

To isolate single-cell suspensions from lung tissue, individual lungs were finely cut and placed in 1 ml of lung digest mix containing 2 U/ml of Liberase (Sigma–Aldrich) and 160 U/ml of DNAseI (Sigma–Aldrich) in Dulbecco’s PBS enriched with magnesium and calcium (Gibco). Lung digests were incubated at 37 °C, shaking at 200 rpm, for 35 min. The Liberase/DNAseI was then inactivated with RPMI (Gibco) containing 10% (v/v) FBS (Thermo Fisher Scientific). Digested lungs were pushed through a 70 μm cell strainer to generate a single-cell suspension, and red blood cells were lysed using ACK lysing buffer (Gibco). Cells were then washed with RPMI containing 10% (v/v) FBS and resuspended at 1 × 10^7^ cells/ml.

### Flow cytometry

BALF-containing cells were centrifuged at 1500 rpm for 5 min at 4 °C. Supernatants were collected for BALF cytokine measurements, and cells were used for analysis by flow cytometry. Single-cell suspensions, diluted in PBS, were used to analyze total cell counts on BD FACSVerse (BD Biosciences). For analysis, cells were washed with fluorescent-activated cell sorting buffer (1% [w/v] bovine serum albumin in PBS), and Fcγ receptors were blocked for 15 min at 4 °C using Mouse BD Fc Block (BD Biosciences). For cultured macrophages, adherent cells were collected by incubation for 5 min with 0.48 mM EDTA in PBS on ice and then scraping to form a single-cell suspension. Cells were stained with fixable viability dye eFluor 450 (eBioscience) or Fixable Blue Live/Dead stain (Thermo Fisher Scientific), prior to incubation with Fc block. Cell surface markers were stained, and data were acquired on either BD FACSCanto II or LSR Fortessa (BD Biosciences) flow cytometers using FACSDiva software (BD Biosciences). Analysis was performed using FlowJo (Tree Star, Inc). Antibodies used for flow cytometry are listed in Table 1, and representative gating strategies are shown in Figs. S4 and S5.

**Table 1:** Antibodies used for flow cytometry

Antigen	Supplier	Clone	Fluorophore
CD45	BioLegend	30-F11	BV510 or AF700
Gr1	BioLegend	RB6-8C5	BV421
CD11b	BioLegend	M1/70	FITC
CD11c	BioLegend	N418	APC
TCRβ	BioLegend	H57-597	PE-Cy5.5
CD4	BioLegend	Gk1.5	PE-Cy7
CD8	BioLegend	Ly-2	PE-Cy7
CD19	BD Biosciences	1D3	APC-H7
CD206	BioLegend	C068C2	APC, PE-Cy7, or PE
CD64	BioLegend	X54-5/7.1	APC
CD301a	BioLegend	LOM-8.7	PE
F4/80	BioLegend	BM8	FITC or BV421
Siglec F	BD Biosciences	E50-2440	PE
Siglec F	BioLegend	S17007L	PE
Ly6G	BioLegend	1A8	PerCP

### Cytokine measurements

Cytokines were measured using Bio-Plex Pro assay system (Bio-Rad Laboratories), Luminex 200 machine, and xPONENT 4.1 software (Luminex Corporation) according to the manufacturer’s protocols.

### Statistical analysis

Statistical analysis was carried out using GraphPad Prism. For analysis of weight loss, the area under/above the curve was calculated relative to the weight on day 0 using GraphPad Prism. The specific statistical tests used are indicated in the figure legends. For ANOVA, significant differences from post hoc testing are indicated on the figures where appropriate. Details of the *F* and *p* values for all ANOVAs are given in the legend (Fig. 1) or supporting tables (Figure 2, Figure 3, Figure 4, Figure 5, Figure 6, Figure 7).

## Data availability

All data relevant to this work are contained within this article and the associated supporting information.

## Supporting information

This article contains supporting information.

## Conflict of interest

T. T. is an employee of Ono Pharmaceutical Co Ltd. All the other authors declare that they have no conflicts of interest with the contents of this article.
