# Supplementary material for: Salt-inducible kinase 2 regulates fibrosis during bleomycin-induced lung injury
Source: J Biol Chem. 2022 Oct 26;298(12):102644. doi: 10.1016/j.jbc.2022.102644 (PMC9706632; doi:10.1016/j.jbc.2022.102644)
Supplement: Supporting information [file mmc1.pdf]

Supporting Information

Salt Inducible Kinase 2 regulates fibrosis during bleomycin induced lung injury.

Manuel van Gijsel-Bonnello[1,2], Nicola J. Darling[2], Takashi Tanaka[3], Samuele Di Carmine[1], Francesco Marchesi[4], Sarah Thomson[5], Kristopher Clark[2], Mariola Kurowska-Stolarska[6], Henry H. McSorley[1], Philip Cohen[2] and J. Simon C. Arthur[1]

1 Division of Cell Signalling and Immunology, School of Life Sciences, University of Dundee, Dundee, DD1 5EH, U.K.
2 MRC Protein Phosphorylation and Ubiquitylation Unit, School of Life Sciences, University of Dundee, Dundee, DD1 5EH, U.K.
3 Research Center of Specialty, Ono Pharmaceutical Co. Ltd., 3-1-1 Sakurai, Shimamoto-cho, Mishima-gun, Osaka 618-8585, Japan.
4 School of Veterinary Medicine, College of Medical Veterinary and Life Sciences, University of Glasgow, Glasgow, G12 8TA, U.K.
5 Biological Services, University of Dundee, Dundee, DD1 5EH, U.K.
6 Institute of Infection, Immunity and Inflammation, College of Medical Veterinary and Life Sciences, University of Glasgow, Glasgow, G12 8TA, U.K.

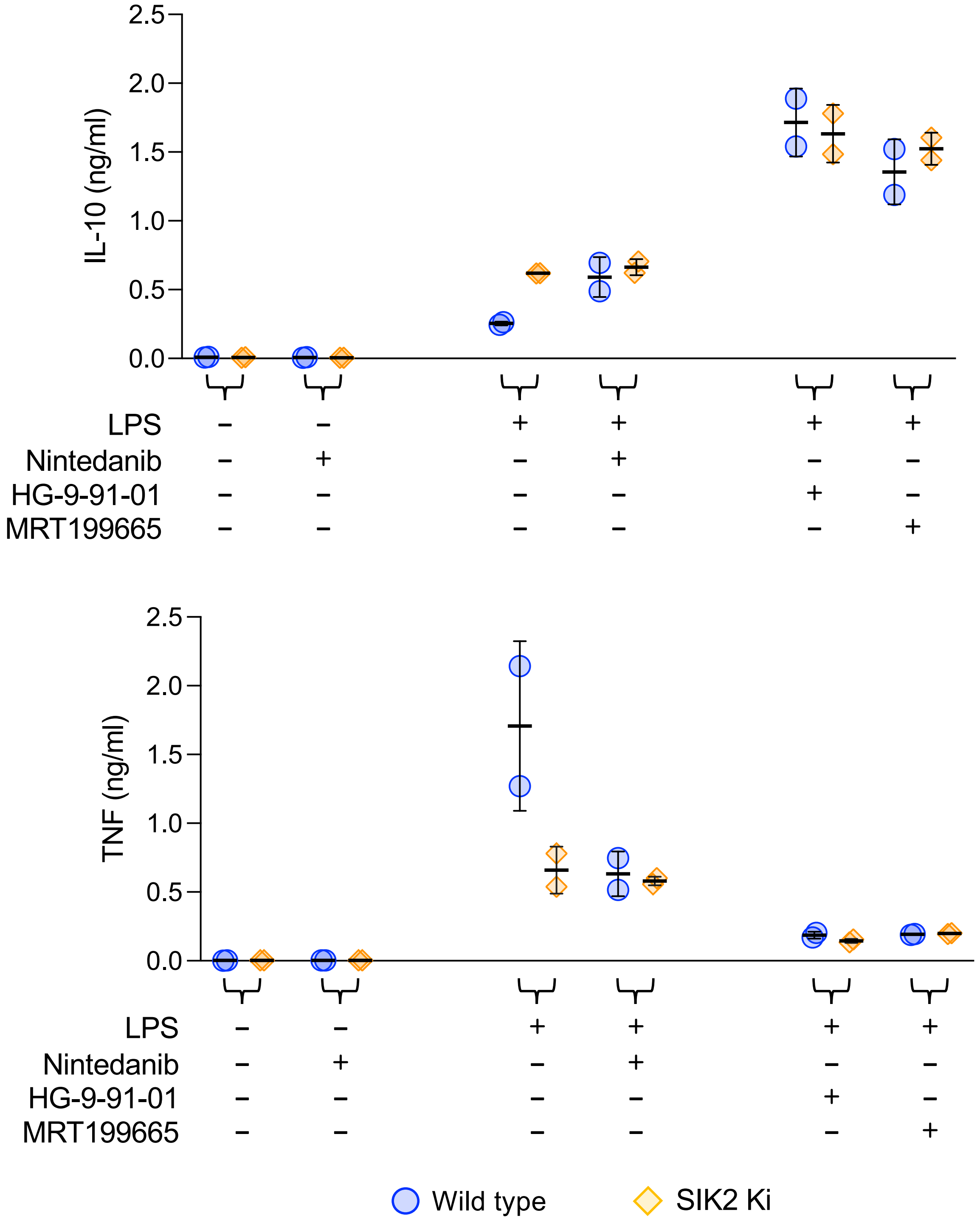

Supplementary figure 1. Effect of Nintedanib on IL-10 and TNF production in LPS stimulated macrophages.
BMDMs were generated from wild type or SIK2 knockin (Ki) mice. Where indicated cells were incubated with 3 μM Nintedanib, 0.5 μM HG-9-91-01 or 1 μM MRT199665 for 1 h before stimulation with 100 ng/ml LPS. Cytokine levels in the media were measured following 2 h LPS stimulation for IL-10 and 6 h for TNF. Data shows mean and standard deviation of 2 mice per genotype with symbols indicating the values for individual mice. For each mouse, data represents the average measurements from 3 wells.

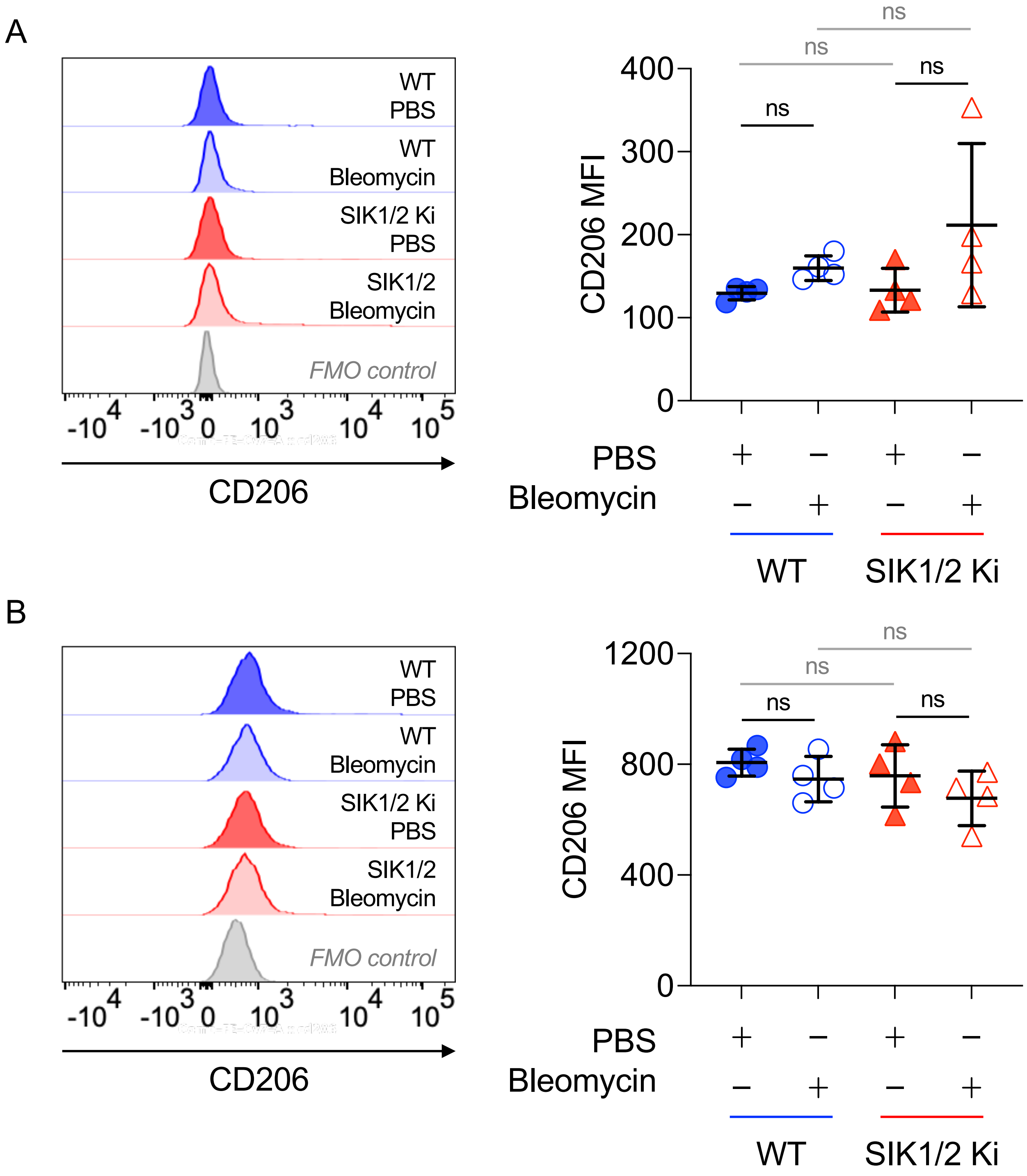

Supplementary figure 2. CD206 expression in lung myeloid cells.
Wild type and SIK1/2 knockin mice were given an oropharyngeal dose of 1.5 mg/kg bleomycin or an equivalent volume of PBS. Mice were culled on day 7 and single cell suspensions prepared from lung digests. Cells were stained with CD45, CD11c, Siglec F, CD206, F4/80, Ly6G and CD11b and analysed by flow cytometry. Expression of CD206 in $CD45^{+ve}/CD11c^{-ve}/CD11b^{+ve}/Ly6G^{-ve}$ myeloid cells (A) and in $CD45^{+ve}/CD11c^{-ve}/CD11b^{+ve}/Ly6G^{+ve}$ neutrophils (B) are shown. Representative histograms for CD206 expression are shown in the left panels and quantification of MFI in the right panels. 4 mice were analysed per group and graphs show mean and standard deviation with individual mice shown by symbols. Data was analysed by two way ANOVA with Sidak's post-hoc testing to compare between PBS and bleomycin (black) or wild type and SIK1/2 knockin genotypes (grey). Ns indicated a $p > 0.05$.

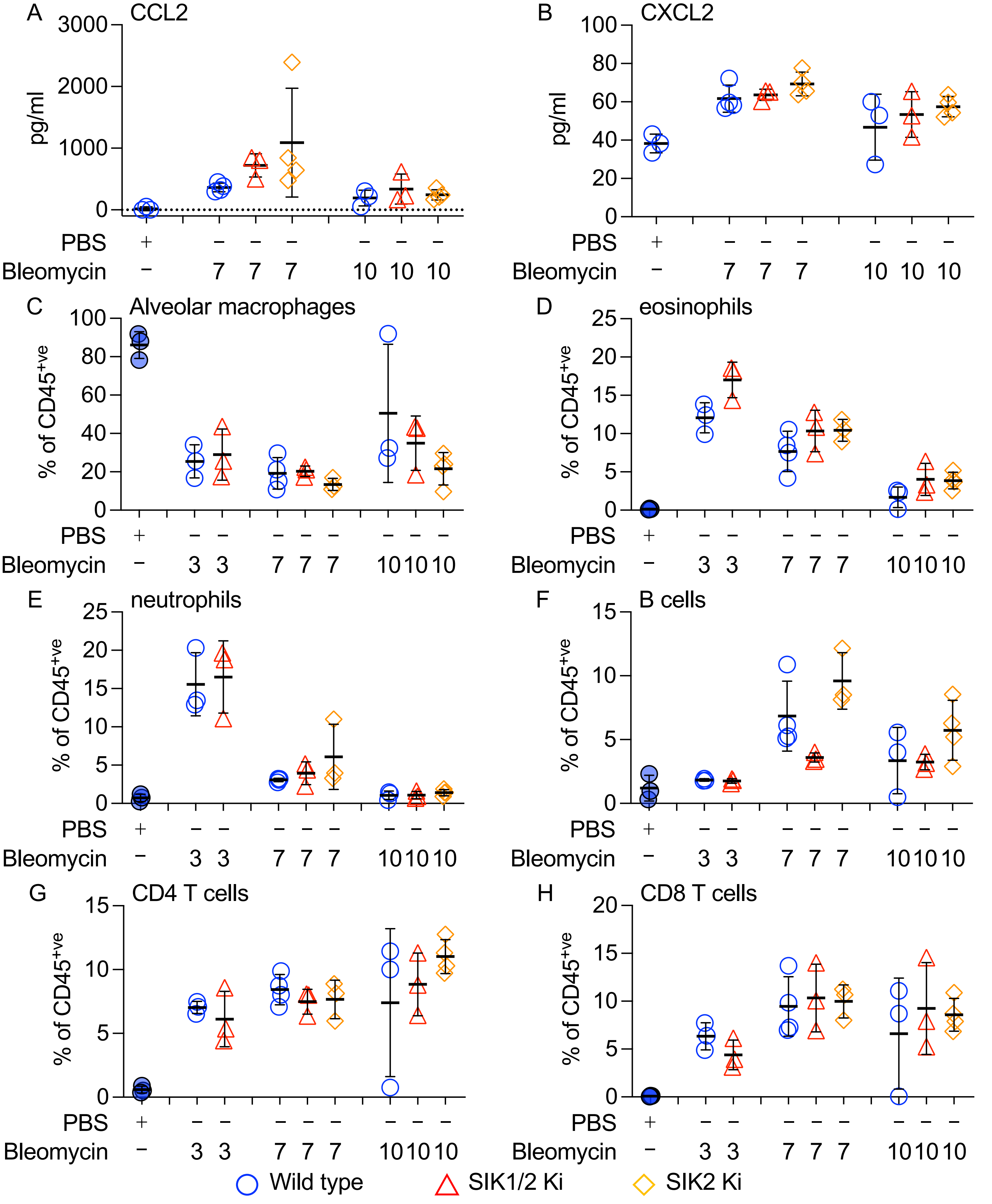

Supplementary figure 3. Cell recruitment and chemokine induction following bleomycin treatment.

Wild type, SIK1/2 or SIK2 knockin (Ki) mice were given an oropharyngeal dose of 2 mg/kg of bleomycin or a PBS control and sacrificed on day 3, 7 or 10. The level of CCL2 (A) and CXCL2 (B) in the BALF was determined. Immune cells in the BALF were analysed by flow cytometry and results for alveolar macrophages (C), eosinophils (D), neutrophils (E), B cells (F), CD4 T cells (G) and CD8 T cells (H) as a percentage of $CD45^{+ve}$ cells are shown. Graphs show mean and standard deviation with symbols representing individual mice. Differences between wild type and SIK1/2 or wild type and SIK2 knockin were not significant at either the 3, 7 or 10 day time points ($p > 0.05$ two way ANOVA and Sidak's post-hoc tests). Gating is shown in supplementary figure 4.

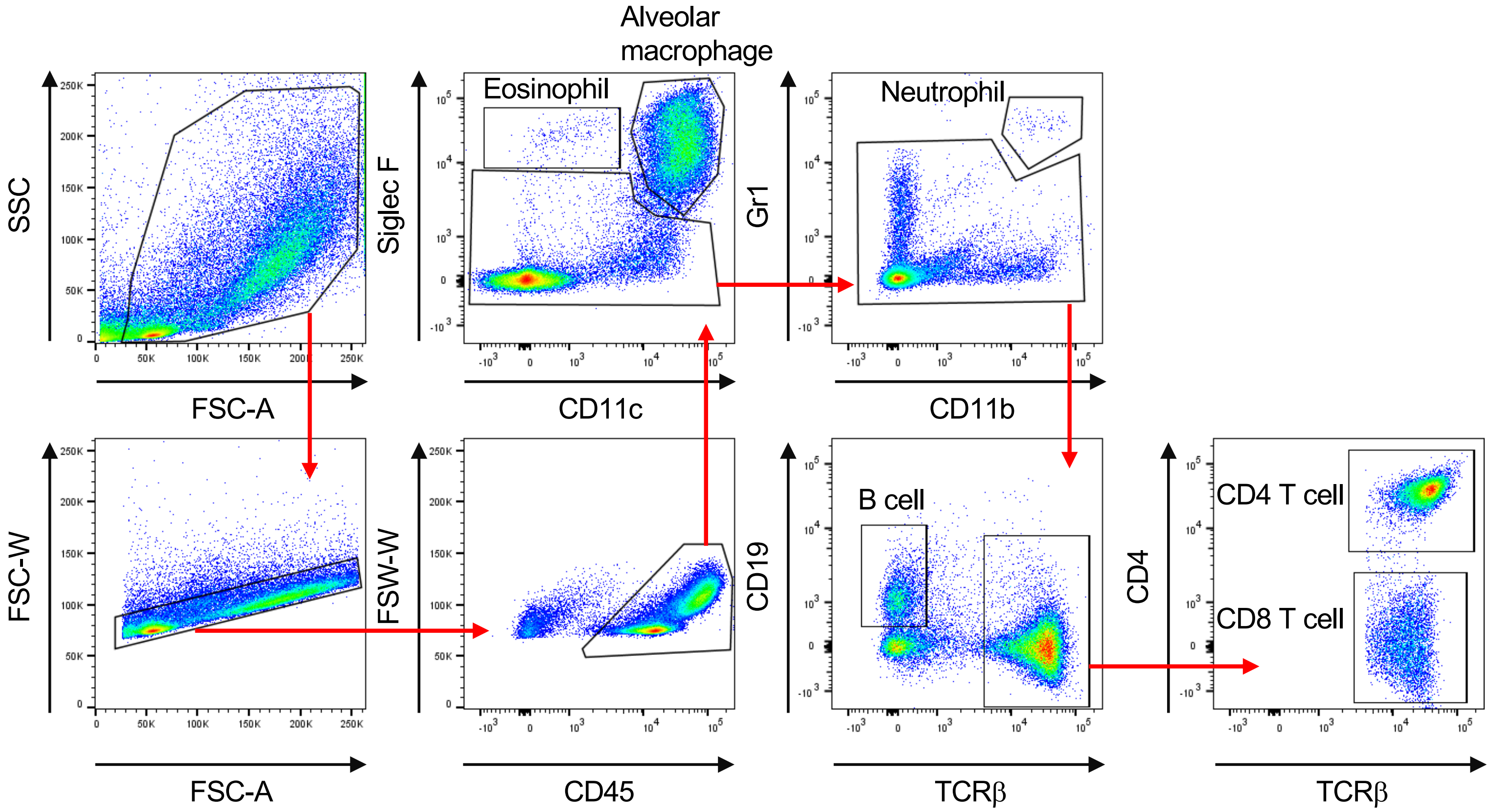

Supplementary figure 4. Gating strategy for the analysis of cells in the BALF by flow cytometry.
Staining of cells in the BALF was carried out with a panel of antibodies comprising of CD45-BV510, CD11c-APC, Siglec F-PE, CD11b-FITC, Gr1-BV421, TCRβ-PE-Cy5.5, CD19-APC-H7 and CD4-PE-Cy7 as described in the methods. The gating strategy used to identify specific immune cell populations. Eosinophils were defined as CD45^{+ve} $^{+ve}$ CD11c^{low}, alveolar macrophages as CD45^{+ve} Siglec F^{+ve} CD11c^{high}, neutrophils as CD45^{+ve} Siglec F^{-ve} CD11b^{+ve} Gr1^{+ve}, B cells as CD45^{+ve} Siglec F^{-ve} CD19^{+ve} TCRβ^{-ve}, CD4 T cells as CD45^{+ve} Siglec F^{-ve} CD19^{-ve} TCRβ^{+ve} CD4^{+ve}, and CD8 T cells as CD45^{+ve} Siglec F^{-ve} CD19^{-ve} TCRβ^{+ve} CD4^{-ve}.

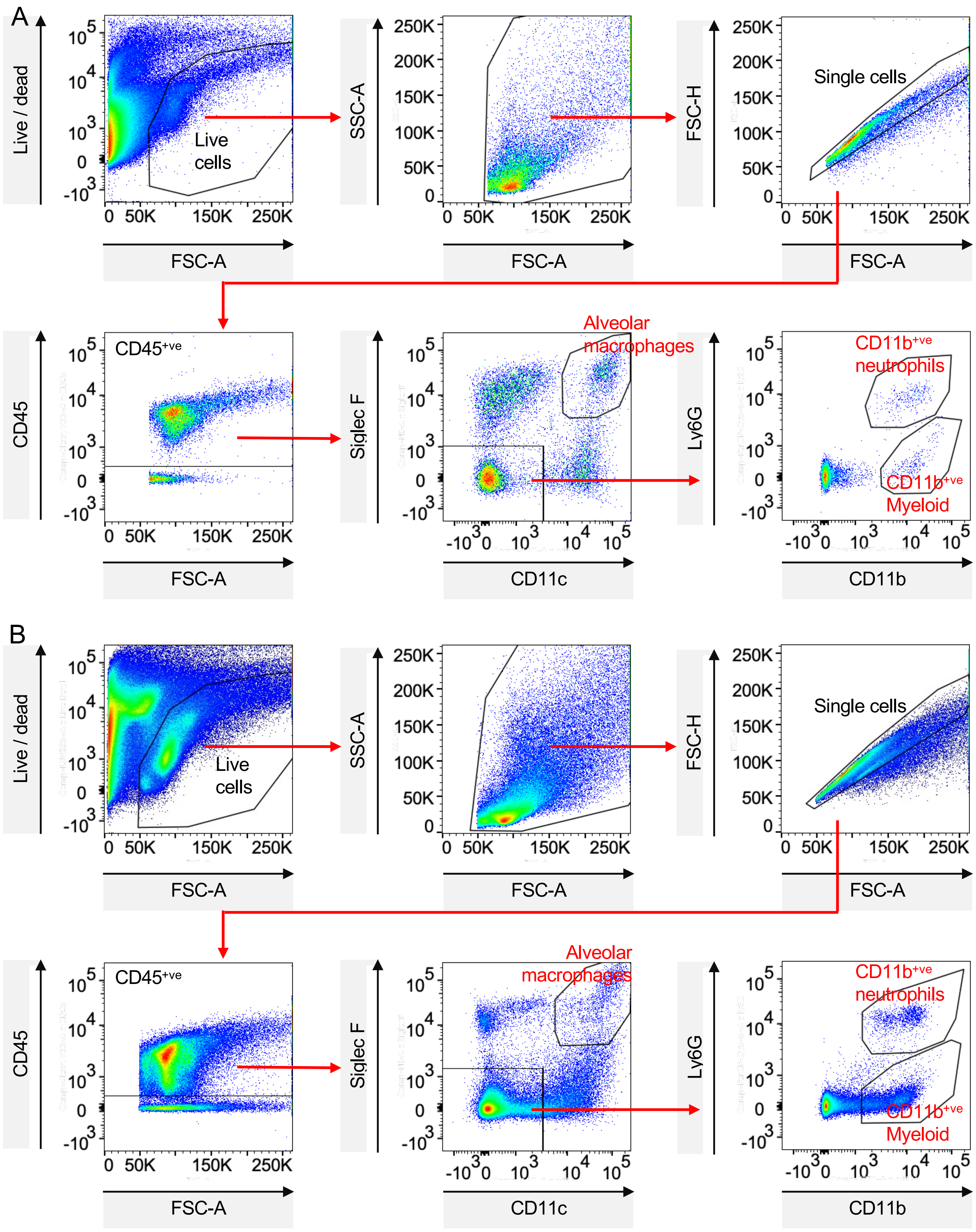

Supplementary figure 5. Gating strategy for the analysis of CD206 expression in the lung

Lung digests were prepared as described in the methods. Cell preps from the BALF (A) or lung (B) were stained with a panel of antibodies comprising of CD45-Alexa700, CD11c-APC, Siglec F-PE, Ly6G-PerCP-Cy5, CD206-PE-Cy7, F4/80-BV421 and CD11b-FITC and a blue Live/dead stain. The gating strategy used to identify alveolar macrophages (CD45^{+ve}/Siglec F^{+ve}/CD11c^{+ve}), CD45^{+ve}/CD11c^{-ve}/CD11b^{+ve}/Ly6G^{-ve} myeloid cells and CD45^{+ve}/CD11c^{-ve}/CD11b^{+ve}/Ly6G^{+ve} neutrophils is shown.

Supplementary table 1. In vitro kinase profiling of Nintedanib

Nintedanib was assayed against 140 kinases at 1 and 0.1 µM as described in the methods. The percentage activity remaining is shown and data represents mean and standard deviation of 2 replicates

Family	kinase	1µM	stdev	0.1µM	stdev
AGC	PKBa	103	9	116	11
AGC	PKCa	94	0	107	8
AGC	PKBb	89	17	98	14
AGC	PKCγ	88	7	97	5
AGC	PKA	94	1	94	6
AGC	PRK2	68	1	93	6
AGC	PKCz	82	7	92	19
AGC	ROCK 2	85	12	92	13
AGC	PDK1	73	27	86	50
AGC	S6K1	53	1	85	0
AGC	MSK1	49	1	77	0
AGC	SGK1	68	16	69	11
AGC	RSK2	50	16	62	12
AGC	RSK1	22	1	57	4
atypical	EF2K	126	21	118	2
CaMK	PRAK	105	12	114	18
CaMK	BRSK1	80	11	111	6
CaMK	PHK	81	3	110	15
CaMK	CHK2	79	6	107	4
CaMK	MARK1	84	10	103	5
CaMK	PIM2	95	7	102	19
CaMK	DAPK1	94	4	102	8
CaMK	MAPKAP-K3	96	1	100	11
CaMK	LKB1	97	0	100	3
CaMK	PIM3	62	4	93	3
CaMK	MARK3	60	1	92	4
CaMK	MARK2	82	1	92	28
CaMK	MNK2	80	13	91	14
CaMK	PIM1	60	2	90	7
CaMK	MAPKAP-K2	96	16	86	5
CaMK	CHK1	35	15	85	31
CaMK	MNK1	88	19	85	13
CaMK	TSSK1	30	3	84	13
CaMK	BRSK2	73	5	82	11
CaMK	CAMK1	51	9	79	34
CaMK	PKD1	67	24	77	19
CaMK	MARK4	78	1	76	20
CaMK	STK33	69	22	76	5
CaMK	SIK3	56	18	76	12
CaMK	SmMLCK	9	1	53	1
CaMK	AMPK	8	0	51	1
CaMK	NUAK1	5	0	35	7
CaMK	SIK2	5	1	16	9
CaMK	MELK	1	0	8	1
CK1	CK1δ	110	5	122	6
CK1	CK1γ2	112	8	114	8
CK1	TTBK1	93	0	102	6
CK1	TTBK2	120	4	91	25
CMGC	p38d	113	7	128	0
CMGC	p38b	79	9	124	25
CMGC	ERK1	130	6	118	23
CMGC	ERK8	98	1	107	0
CMGC	SRPK1	101	1	106	5
CMGC	JNK1	90	3	106	6
CMGC	JNK2	84	6	106	3
CMGC	ERK2	100	9	105	11
CMGC	DYRK2	99	4	104	2
CMGC	JNK3	102	14	103	11
CMGC	CLK2	86	1	102	6
CMGC	DYRK3	100	11	101	9
CMGC	p38g	83	8	101	5
CMGC	HIPK3	93	1	99	6
CMGC	HIPK2	73	11	93	9
CMGC	p38a	80	13	90	10
CMGC	HIPK1	97	2	85	20
CMGC	CDK9-CyclinT1	76	24	82	9
CMGC	GSK3b	32	3	80	2
CMGC	CDK2-Cyclin A	89	16	79	35
CMGC	DYRK1A	87	23	78	3
CMGC	ERK5	63	0	70	8

Family	kinase	1µM	stdev	0.1µM	stdev
other	NEK6	116	3	114	9
other	PINK	108	11	110	4
other	EIF2AK3	101	5	108	12
other	TTK	84	2	107	2
other	CK2	98	10	106	4
other	ULK1	94	3	106	10
other	MPSK1	93	6	104	22
other	Aurora A	71	7	103	0
other	CAMKKb	69	4	99	0
other	TLK1	92	3	99	9
other	WNK1	99	9	97	11
other	ULK2	82	4	92	34
other	PLK1	79	5	86	10
other	Aurora B	19	1	74	1
other	NEK2a	76	2	73	0
other	IKKb	77	10	66	5
other	IKKe	19	5	34	1
other	TBK1	20	6	59	5
STE	MEKK1	99	16	113	8
STE	GCK	74	9	106	10
STE	TAO1	99	2	105	2
STE	MKK2	58	2	101	6
STE	PAK6	98	8	99	4
STE	PAK4	73	5	91	10
STE	ASK1	78	1	82	6
STE	PAK5	92	1	80	1
STE	OSR1	76	17	79	1
STE	MAP4K5	30	2	76	4
STE	MKK6	79	4	76	2
STE	MST4	48	1	76	1
STE	PAK2	91	21	75	32
STE	MST2	26	6	71	16
STE	MKK1	43	6	71	6
STE	MINK1	19	5	66	20
STE	MAP4K3	8	1	59	2
STE	MST3	14	0	57	10
TK	IGF-1R	82	4	120	30
TK	HER4	88	2	111	8
TK	ZAP70	81	8	104	11
TK	EPH-A2	58	14	104	2
TK	CSK	53	4	98	5
TK	SYK	65	4	96	2
TK	EPH-B4	39	1	95	5
TK	IR	52	2	94	13
TK	EPH-B1	36	10	85	0
TK	TIE2	53	10	81	14
TK	EPH-B3	63	5	69	20
TK	EPH-A4	51	5	66	14
TK	IRR	26	1	57	6
TK	EPH-B2	29	8	56	26
TK	BRK	21	1	53	21
TK	FGFR1	10	3	48	7
TK	DDR2	27	7	39	8
TK	BTK	38	1	37	12
TK	VEGFR	6	3	35	1
TK	JAK2	6	0	31	0
TK	Src	4	0	28	4
TK	ABL	5	1	25	0
TK	PDGFRA	5	0	7	1
TK	TrkA	3	0	7	1
TK	Lck	3	0	4	0
TK	YES1	1	2	3	3
TKL	MLK1	44	5	89	6
TKL	MLK3	32	3	77	1
TLK	TGFBR1	101	4	117	12
TLK	IRAK1	83	3	105	9
TLK	IRAK4	75	1	91	1
TLK	RIPK2	29	7	89	27
TLK	TESK1	73	1	70	5
TLK	TAK1	17	4	39	0

Supplementary table 2. Histology scoring scales

Fibrosis

Fibrosis is scored in 15 microscopic fields (in MT stained section) at x200 magnification according to the following ordinal grading scale

Score	Definition
0	No fibrosis / tissue within normal limits
1	minimal accumulation of fibrous tissue in separate alveolar septa and/or mild focal subpleural fibrosis
2	mildly increased amounts of fibrous tissue in adjacent alveolar septa and/or more prominent patchy subpleural fibrosis
3	moderate increase in fibrous tissue adjacent alveolar septa with retained alveolar architecture and/or moderate focally extensive subpleural fibrosis
4	locally extensive coalescing areas of marked fibrous thickening of alveolar septa (>60% of the microscopic field) with variable obliteration of alveolar lumens

Inflammation

Inflammatory lesions are scored in 15 microscopic fields (in HE stained section) at x200 magnification according to the following ordinal grading scale

Score	Definition
0	tissue within normal limits (no inflammation or only minimal presence of intra-alveolar macrophages)
1	mild interstitial and/or alveolar inflammatory infiltrates affecting <20% of the lung tissue within a x200 microscopic field
2	moderate multifocal interstitial and/or alveolar inflammatory infiltrates affecting 20-50% of the lung tissue within a x200 microscopic field
3	marked multifocal to coalescing interstitial and/or alveolar inflammatory infiltrates affecting 50-70% of the lung tissue within a x200 microscopic field
4	severe extensive to diffuse interstitial and/or alveolar inflammatory infiltrates affecting >70% of the lung tissue within a x200 microscopic field

Type II pneumocyte hypertrophy/hyperplasia

Hypertrophy/hyperplasia of type II pneumocytes is scored in 15 microscopic fields (in HE stained section) at x200 magnification according to the following ordinal grading scale

Score	Definition
0	tissue is within normal limits
1	minimal hypertrophy/hyperplasia affecting single or scattered alveolar spaces (<10% of the microscopic field)
2	mild hypertrophy/hyperplasia affecting multiple alveolar spaces (10-30% of the microscopic field)
3	moderate hypertrophy/hyperplasia affecting focally extensive or multifocal areas of alveolar spaces (30-50% of the microscopic field)
4	marked hypertrophy/hyperplasia affecting extensive and coalescing areas of alveolar spaces (>50% of the microscopic field)

Supplementary table 3. Statistical testing of Figure 2.

F and *p* values from two way ANOVAs for data plotted in Figures 2A, B and C.

Figure	Data	Genotype F	*p*	Treatment F	*p*	Interaction F	*p*
Fig 2A	% CD64+ve	25.24	<0.0001	17.37	<0.0001	3.313	0.0595
	CD64 MFI	25.59	<0.0001	179.6	<0.0001	8.117	0.0031
Fig 2B	% CD206+ve	109.8	<0.0001	26.15	<0.0001	6.121	0.0094
	CD206 MFI	41.91	<0.0001	25.36	<0.0001	0.1442	0.8667
Fig 2C	% CD301+ve	10.95	0.0039	488.1	<0.0001	11.99	0.0005
	CD301 MFI	9.252	0.007	336.1	<0.0001	6.274	0.0086

Supplementary table 4. Statistical testing of Figure 3.

F and *p* values from two way ANOVAs for data on alveolar macrophages plotted in Figures 3C to F. Values for the $CD45^{+ve}/CD11c^{-ve}/CD11b^{+ve}/Ly6G^{-ve}$ myeloid cells and $CD45^{+ve}/CD11c^{-ve}/CD11b^{+ve}/Ly6G^{+ve}$ neutrophils from the same experiment, that is presented in Supplementary Figure 2, are also given.

Figure	Data	Genotype F	*p*	Treatment F	*p*	Interaction F	*p*
Fig 3C	CD206 MFI BAL	1.931	0.188	118.9	<0.0001	1.557	0.2342
Fig 3D	CD206 MFI lung	0.8092	0.386	89.68	<0.0001	0.1283	0.7264
Fig 3E	% Av. Macrophages BAL	0.3013	0.5924	202.1	<0.0001	0.8372	0.3769
Fig 3F	% Av. Macrophages lung	2.003	0.1824	52.56	<0.0001	0.9047	0.3603
Sup Fig 2A	CD206 MFI Meyloid	1.277	0.2805	4.266	0.0612	0.786	0.3927
Sup Fig 2B	CD206 MFI Neutrophils	1.392	0.2609	4.522	0.0549	0.3653	0.5568

Supplementary table 5. Statistical testing of Figure 4.

F and *p* values from two way ANOVAs for data on the comparisons between wild type and knockin mice on days 7 and 10 shown in the graphs in Figure 4. Data from the same experiment was also shown in Supplementary Figure 3. For the cell numbers in the supplementary figure, separate ANOVAs were used to compare wild type and SIK2 knockin mice at days 3, 7 and 10 and wild type and SIK1/2 knockin mice at days 7 and 10 only.

Figure	Data	Genotype F	*p*	Treatment F	*p*	Interaction F	*p*
Fig 4A	TGF	3.593	0.0531	3.855	0.0684	0.432	0.6571
Fig 4B	IL-22	1.878	0.1872	0.4974	0.4914	1.118	0.3527
Fig 4C	TNF	0.6783	0.5224	0.02001	0.8894	0.5327	0.5977
Fig 4D	IL-10	0.421	0.6639	1.868	0.1919	0.1893	0.8295
Fig 4E	GM-CSF	2.046	0.1638	1.491	0.2409	0.9229	0.4188
Fig 4F	CD45+ve cells	7.609	0.0065	53.63	<0.0001	2.469	0.1234
Sup Fig 3A	CCL2	1.638	0.2273	6.478	0.0224	1.286	0.3052
Sup Fig 3B	CXCL2	1.882	0.1866	9.487	0.0076	0.1086	0.8978
Sup Fig 3C	Av. Macrophages WT vs SIK2	0.2197	0.647	3.034	0.0829	0.5767	0.5755
Sup Fig 3C	Av. Macrophages WT vs SIK1/2	3.403	0.0949	4.42	0.0618	1.519	0.246
Sup Fig 3D	Eosinophils WT vs SIK2	10.15	0.0072	40.17	<0.0001	0.6035	0.5615
Sup Fig 3D	Eosinophils WT vs SIK1/2	6.552	0.0284	42.31	<0.0001	0.09026	0.77
Sup Fig 3E	Neutrophils WT vs SIK2	0.2744	0.6092	60.51	<0.0001	0.06206	0.9401
Sup Fig 3E	Neutrophils WT vs SIK1/2	2.569	0.1401	10.16	0.0097	1.628	0.2308
Sup Fig 3F	B cells WT vs SIK2	2.134	0.1678	6.648	0.0103	1.903	0.1884
Sup Fig 3F	B cells WT vs SIK1/2	3.624	0.0861	7.435	0.0213	0.01919	0.8926
Sup Fig 3G	CD4 T cells WT vs SIK2	0.0127	0.912	0.602	0.5623	0.3897	0.6849
Sup Fig 3G	CD4 T cells WT vs SIK1/2	0.8558	0.3767	0.5737	0.4663	2.025	0.1852
Sup Fig 3H	CD8 T cells WT vs SIK2	0.09487	0.763	2.425	0.1273	0.5875	0.5698
Sup Fig 3H	CD8 T cells WT vs SIK1/2	0.4796	0.5044	1.383	0.2668	0.1616	0.6962

Supplementary table 6. Statistical testing of Figure 5.

Wild type (WT), SIK1/2 knockin (Ki) or SIK2 knockin mice were given an oropharyngeal dose of 2 mg/kg of bleomycin (Bleo) or PBS as a control. Weight was monitored daily for 22 days. Mice which lost more than 20% of their body weight without showing signs of improvement within 3 days were considered to have reached the humane endpoint and were sacrificed at that point. The average percentage weight change relative to the weight on day -1 before bleomycin treatment is shown in Figure 5C and F. The tables show the results of a Mixed Effect Model analysis followed by Tukey's post-hoc testing to determine significance between experimental groups on individual days. A $p < 0.05$ is indicated by *, < 0.01 by **, < 0.001 by *** and < 0.0001 by ****. Non significant ($p > 0.05$) are indicated by ns. Area under the curve was also calculated (Fig 5B, E) and analysed by two-way ANOVA. F and p values for this are given in the lower tables.

Mixed Model analysis

Comparison (Fig 5C)	F	*p*
Time	9.291	<0.0001
Genotype	17.26	<0.0001
Interaction	4.402	<0.0001

Tukey's post-hoc testing

Comparison (Fig 5C)	main effect	Day -1	0	1	2	3	4	5	6	7	8	9	10	11	12	13	14	15	16	17	18	19	20	21	22
WT PBS vs WT Bleo	<0.0001	ns	ns	ns	ns	**	***	****	****	****	****	****	****	****	****	****	****	****	****	****	****	****	***	****	****
WT PBS vs SIK2 Ki PBS	0.9984	ns	ns	ns	ns	ns	ns	ns	ns	ns	ns	ns	ns	ns	ns	ns	ns	ns	ns	ns	ns	ns	ns	ns	ns
WT PBS vs SIK2 Ki Bleo	0.0192	ns	ns	ns	ns	ns	ns	ns	*	***	***	**	*	*	ns	ns	*	**	*	ns	ns	ns	ns	ns	ns
WT Bleo vs SIK2 Ki PBS	0.0005	ns	ns	ns	ns	ns	ns	*	**	****	****	****	***	****	****	****	**	***	****	****	****	****	****	***	***
WT Bleo vs SIK2 Ki Bleo	0.0131	ns	ns	ns	ns	ns	ns	*	*	**	**	**	**	*	**	**	ns	ns	*	**	**	**	ns	*	*
SIK2 Ki PBS vs SIK2 Ki Bleo	0.0625	ns	ns	ns	ns	ns	ns	ns	ns	ns	*	ns	ns	*	*	*	ns	ns	*	*	ns	*	*	ns	ns

Mixed Model analysis

Comparison (Fig 5F)	F	*p*
Time	11.72	<0.0001
Genotype	9.667	0.0005
Interaction	3.264	<0.0001

Tukey's post-hoc testing

Comparison (Fig 5F)	main effect	Day -1	0	1	2	3	4	5	6	7	8	9	10	11	12	13	14	15	16	17	18	19	20	21	22
WT PBS vs WT Bleo	0.0008	ns	ns	ns	ns	*	***	****	****	****	****	****	***	***	****	****	***	***	***	****	****	***	**	**	**
WT PBS vs SIK1/2 Ki PBS	0.9418	ns	ns	ns	ns	ns	ns	ns	ns	ns	ns	ns	ns	ns	ns	ns	ns	ns	ns	ns	ns	ns	ns	ns	ns
WT PBS vs SIK1/2 Ki Bleo	0.3716	ns	ns	ns	ns	ns	ns	ns	ns	ns	*	ns	ns	ns	ns	ns	ns	ns	ns	ns	ns	ns	ns	ns	ns
WT Bleo vs SIK1/2 Ki PBS	0.0259	ns	ns	ns	ns	ns	ns	*	*	***	**	*	ns	*	**	**	ns	ns	*	***	**	*	ns	*	ns
WT Bleo vs SIK1/2 Ki Bleo	0.0043	ns	ns	ns	ns	ns	ns	**	**	****	***	**	*	**	***	***	***	***	****	***	***	****	**	**	*
SIK1/2 PBS vs SIK1/2 Ki Bleo	0.9129	ns	ns	ns	ns	ns	ns	ns	ns	ns	ns	ns	ns	ns	ns	ns	ns	ns	ns	ns	ns	ns	ns	ns	ns

Two way ANOVA

WT vs SIK2 Ki (Fig 5B)	F	*p*
Treatment	35.83	<0.0001
Genotype	2.258	0.1465
Interaction	1.423	0.2451

WT vs SIK1/2 Ki (Fig 5E)	F	*p*
Treatment	11.05	0.0038
Genotype	1.415	0.2496
Interaction	5.024	0.0378

Supplementary table 7. Statistical testing of Figure 6.

Figure 6 shows the levels of fibrosis, inflammation and remodelling was assessed on day 22 following bleomycin treatment in wild type, SIK2 knockin and SIK1/2 knockin mice. Differences between wild type and SIK2 knockin or wild type and SIK1/2 knockin mice were analysed by two way ANOVA. F and *p* values form this are shown below.

Figure	Data	Genotype F	*p*	Treatment F	*p*	Interaction F	*p*
Fig 6B	Fibrosis WT / SIK2 Ki	3.15	0.0929	23.05	0.0001	3.15	0.0929
Fig 6C	Fibrosis WT / SIK1/2 Ki	4.373	0.0528	20.28	0.0004	4.373	0.0528
Fig 6E	Inflammation WT / SIK2 Ki	3.165	0.0921	57.61	<0.0001	2.865	0.1078
Fig 6F	Inflammation WT / SIK1/2 Ki	4.036	0.0617	23.8	0.0002	3.091	0.0978
Fig 6G	Remodelling WT / SIK2 Ki	1.684	0.2108	5.696	0.0282	1.38	0.2554
Fig 6H	Remodelling WT / SIK1/2 Ki	0.1767	0.6798	3.046	0.1001	0.09671	0.7598

Supplementary table 8. Statistical testing of Figure 7.

Wild type Vav-iCre^{+ve} or immune cell specific SIK3$^{fl/fl}$/ Vav-iCre^{+ve} knockout (Ko) mice were given an oropharyngeal dose of 1.5 mg/kg of bleomycin or a PBS control. Weight was monitored daily for 20 days. Mice which lost more than 20% of their body weight without showing signs of improvement in 3 days were considered to have reached the humane endpoint and were sacrificed. The average percentage weight change relative to the weight on day 0 before bleomycin treatment is shown in Fig 7B. The tables show the results of a Mixed Effect Model analysis followed by Tukey's post-hoc testing to determine significance between experimental groups on individual days. A $p < 0.05$ is indicated by *, < 0.01 by **, < 0.001 by *** and < 0.0001 by ****. Non significant ($p > 0.05$) are indicated by ns. Area under the curve for the weight change was also calculated (Fig 7C) while inflammation and fibrosis were assessed by histology at day 20 (Fig 7D and E). Data in Fig 7C to E was analysed by two way ANOVA, F and *p* values for this are given in the lower table.

Mixed Model analysis

Comparison (Fig 7B)	F	*p*
Time	3.992	<0.0001
Genotype	8.649	0.0005
Interaction	2.974	<0.0001

Turkey's post-hoc testing

Comparison (Fig 7B)	main effect	0	1	2	3	4	5	6	7	8	9	10	11	12	13	14	15	16	17	18	19	20
WT PBS vs. WT Bleo	0.0032	ns	ns	ns	ns	ns	*	**	***	***	***	***	****	***	***	**	**	***	***	***	**	*
WT PBS vs. SIK3 Ko PBS	0.998	ns	ns	ns	ns	ns	ns	ns	ns	ns	ns	ns	ns	ns	ns	ns	ns	ns	ns	ns	ns	ns
WT PBS vs. SIK3 Ko Bleo	0.0136	ns	ns	ns	ns	ns	ns	*	**	**	**	***	***	**	**	*	*	**	**	**	*	*
WT Bleo vs. SIK3 Ko PBS	0.0055	ns	ns	ns	ns	ns	*	**	***	***	****	***	***	***	**	*	***	**	**	**	*	*
WT Bleo vs. SIK3 Ko Bleo	0.847	ns	ns	ns	ns	ns	ns	ns	ns	ns	ns	ns	ns	ns	ns	ns	ns	ns	ns	ns	ns	ns
SIK3 Ko PBS PBS vs. SIK3 Ko Bleo	0.0225	ns	ns	ns	ns	ns	ns	*	*	**	**	***	***	**	*	*	**	*	ns	*	ns	*

Two way ANOVA

Figure	Data	Genotype F	*p*	Treatment F	*p*	Interaction F	*p*
Fig 7C	AUC (weight)	0.01199	0.9137	26.13	<0.0001	0.06409	0.8023
Fig 7D	Inflammation	0.01341	0.9092	22.69	0.0002	0.1316	0.7215
Fig 7E	Fibrosis	0.2717	0.6093	22.15	0.0002	0.2717	0.6093
